# The Research Focus of Nations: Economic vs. Altruistic Motivations

**DOI:** 10.1371/journal.pone.0169383

**Published:** 2017-01-05

**Authors:** Richard Klavans, Kevin W. Boyack

**Affiliations:** 1 SciTech Strategies, Inc., Wayne, PA, United States of America; 2 SciTech Strategies, Inc., Albuquerque, NM, United States of America; Universidad Veracruzana, MEXICO

## Abstract

What motivates the research strategies of nations and institutions? We suggest that research primarily serves two masters–altruism and economic growth. Some nations focus more research in altruistic (or non-economic) fields while others focus more research in fields associated with economic growth. What causes this difference? Are there characteristics that would suggest why a nation is more aligned with altruism or economic growth? To answer this question, we have identified nine major fields of research by analyzing the publication activity of 4429 institutions using Scopus data. Two fields of research are clearly altruistic (there is relatively little involvement by industry) and two fields are clearly aligned with economic growth. The altruistic vs. economic nature of nations based on their publication profiles across these fields is correlated with national indicators on wealth, education, capitalism, individualism, power, religion, and language. While previous research has suggested that national research strategy is aligned with national wealth, our analysis shows that national wealth is not highly correlated with the tradeoff between altruistic and economic motives. Instead, the tradeoff is largely captured by a culture of individualism. Accordingly, implications for national research strategies are discussed.

## Introduction

Much of the current focus of research in the United States can trace its roots back to altruistic goals elegantly stated in “Science: The Endless Frontier” [1], one of whose primary focal points was on medical advances that would benefit the world. Relative investment in medical research has increased around the world since that time. For example, the funding for health-related R&D in the U.S. has risen from 15% to over 50% of the non-defense R&D budget from 1953 to 2013 [2]. One consequence of this long-term research investment and the corresponding research investments from many nations is that the average life expectancy of a newborn child has increased from 46.8 years in 1950 to 70.5 years in 2015 [3]. Medical research continues to evolve as progress is made in diagnosing and treating disease.

It is somewhat surprising, given this history, that altruism is not explicitly acknowledged in research policy. Rather, the dominant perspectives on ‘why a nation should spend money on research’ are rooted in theories of innovation and economic growth. Correspondingly, the focus is on inventors and entrepreneurs who create and exploit scientific and technical opportunities. In general, the economic view of science, invention, innovation and growth does not incorporate what one observes every day–the extensive personal commitment to altruistic causes such as health and well-being, education, mitigating threats to the environment, and creating better communities and societies in which to live. Such commitment, although it starts with individuals, is manifest at the national level as the sum of personal efforts that become highly visible and reflect national values.

It is well known that scientific research portfolios vary widely from nation to nation. What is not yet known is why these portfolios vary. Much of the previous work to address this question has implicitly focused on the economic motive for research. For example, the well-known work of both May [4] and King [5] focused on productivity and wealth as primary explanations for a national strategy of research intensity.

Our intent in this study is to explore why nations support research with non-economic characteristics. While we readily acknowledge that there are many different motives for research (e.g., economic, safety, health, quality of life, etc.), and that motives can be intertwined (e.g., reducing illness is both a quality of life issue and highly profitable economically), for purposes of starting a discussion on this topic we choose to represent national research motives as a single continuum between economic motives and altruistic (or non-economic) motives. Specifically, we address the following questions. What are the major research strategies that are being pursued in the world today? Which (and how much) of these activities are more aligned with altruistic motives, and which are more aligned with economic motives? Do the dramatic differences in the research portfolios of nations along the motivation continuum correlate with other national characteristics?

This paper is organized as follows. We start with a discussion of May and King, and other studies that have sought to identify national research strategies. We also discuss the literature surrounding why nations pursue research, with separate discussions of economic and altruistic perspectives. Methods for this study are then introduced, including a new method to identify fields from a publication database for the purpose of identifying national focus. We then expand upon possible reasons for national differences by considering wealth, level of education, form of government (capitalism), culture (individualism, power), religion, and language.

This is followed by a description of our data and results. We make extensive use of the Scopus database in this study, using a discipline-level classification system based on clustering of papers (rather than journals) into 114 disciplines using direct citation analysis. Factor analysis using the disciplinary profiles of 4429 institutions results in the identification of nine fields. The relative participation of industry by field allows us to determine the degree to which each field is economically oriented. The three fields that are most altruistic focus on civics, medical treatment and infectious disease. The three fields that are most aligned with economic motives are all application oriented, and are based around engineering, computing, and applied (micro- and nano-scale) physics. Nation-level indicators as mentioned above are used to explore why nations may focus their research in economic or altruistic fields.

After describing the limitations of this study, we summarize our primary findings. We point out that altruism is the fundamental basis for characterizing the differences between the top two publishing nations (the United States and China) and major geographic regions. This calls into question how nations whose research is more aligned with altruistic motives (US, Great Britain, Australia and the Netherlands) can successfully compete against those nations that focus their efforts on fields associated with economic gain (China, Korea, Taiwan and Russia). China has already reached the #1 publication position in one of the non-altruistic fields. Will that leadership correspondingly translate into innovation and economic growth?

## Background

### Identifying National Research Strategies

This study builds upon a stream of research where publication data is used to detect national research strategies. As such, it is important to start with the seminal studies by May and King. May [4], showed that nations with larger R&D investments had larger shares of the scientific pie in terms of both paper and citation counts. Several years later, King [5] expanded upon May’s study, including a number of R&D funding variants (e.g., HERD, etc.) and extending the analysis to seven broad scientific fields. While largely affirming May’s results and showing the relationship between economic and scientific wealth, he also showed some differences between European countries based on their publication profiles across fields.

It is important to point out that these studies did not examine national research strategies per se. Rather, their focus was on national strengths based on research outcomes (publications and citations). These outcome data were normalized so that national strengths (outcomes greater than the norm) could be determined. Alternative normalization methods have been proposed which correspondingly result in a different ordering of national strengths. For instance, Leydesdorff & Zhou [6], using King’s data, identified a new group of emerging nations with high growth that were not highlighted by King. Rousseau & Rousseau [7] investigated the efficiency of European nations with GDP, R&D expenditures and population as normalizing inputs, and showed that rankings change somewhat with changes in the definition of efficiency. Cole & Phelan [8] showed that when normalized by population, productivity was no longer fully explained by wealth, but that religion, decentralization and competitiveness were also factors. Pan et al. [9] correlated country-level data on cites per paper (CPP) with R&D expenditure per researcher, finding that the correlation was threshold-dependent. Below $100,000 USD per researcher per year there is a strong correlation between CPP and spending, while above that level there is no correlation. Cimini et al. [10], using Scopus data, found that leading nations have more diverse research systems than nations whose research systems are “under construction”.

For purposes of discussion we will refer to these national strengths as national strategies. Nations do not maintain research strengths unless there is an intention to do so. For example, King’s observation that the United Kingdom had very high impact in the medical sciences (in relation to a peer group of nations) reflects a decision to spend a greater percentage of research dollars on medical research. The decision to spend relatively more research dollars on medical research can be interpreted as a national strategy, focusing more national efforts towards this end.

The above-referenced studies were all limited in the sense that they did not attempt to determine the axes of national focus or research strategy and the reasons behind the differences. Table 1 lists five studies that did attempt to answer the strategy question rather than simply looking at strengths. Dore and colleagues [11, 12] were the first to use a more sophisticated method for detecting national research strategies from large scale publication data. Using a 12-year set of publications from the Science Citation Index compiled into 18 high level (journal-based) fields, they used correspondence factor analysis to group fields using the data from 48 countries. They identified a large number of factors, but decided to focus on the first two factorial axes (these two factors had much higher eigenvalues). They mapped the location of nations on these two axes. Their first axis highlighted the differences between the natural (labeled by Doré as ‘ancient’) and life (or ‘modern’) sciences, while the second axis differentiates between agricultural sciences and geosciences. They found that nations occupied all four quadrants of their graph, and concluded that nations were choosing between four distinct research strategies.

**Table 1:** Research strategies as identified by studies of national publication patterns.

Study	Data	Strategies	Strategy descriptors
Doré 1996, 2001	SCI 1981–1992	4	Natural; Life; Agriculture; Geo
REIST-2 1997		4	Life; Natural; Engineering; Bio-Env
Schulz 2012	Science Watch	4	Roughly concur with REIST-2
Moya 2013	Scopus 1996–2006	3	BioMed, Basic S&E; Agriculture
Chen 2016	SCI 1994–2011	3	Medical, Natural, Developing

Our second example comes from the *Second European Report on S&T Indicators 1997 (REIST-2)* [13], one of whose analyses was to determine preferred research fields for scientific collaboration by country. Fields were grouped, and four publication profile patterns were identified: 1) a ‘western model’ based around clinical medicine and biomedical research, 2) a ‘former communist model’ focused on chemistry and physics, 3) a ‘Japanese model’ centered on engineering and chemistry, and 4) a ‘bio-environmental model’ with a focus on biology, earth and space sciences. Schulz & Manganote [14] used Science Watch (Thomson Reuters) country profile data and found patterns similar to those from the REIST-2 report. One novel feature of this study was that it included the social sciences, and that this inclusion differentiated England from most other European nations. Moya-Anegón & Herrero-Solana [15] also included the social sciences, although these data did not figure strongly into the results, which featured three clusters of nations–the biomedical cluster, the basic science and engineering cluster, and the agriculture cluster. Finally, Chen & Chen [16] grouped 100 nations into 12 groups using minimum spanning trees, finding that nations within each group were similar in terms of geography, ethnicity, or economic status. Further clustering placed nine of those groups into three main clusters–a Western cluster focused on biomedicine, an Asian and East European cluster focused on the natural sciences, and a third cluster associated with developing countries.

At the level of three or four major research strategies the results from these five studies are relatively robust. All five contain two clusters focused on the natural sciences (i.e. chemistry, physics, etc.) and life sciences (i.e. biomedicine), while three contain a third cluster focused on agriculture that is associated with developing countries. The use of factor analysis, or other forms of dimensional reduction, enables identification of the different research strategies that a nation can pursue.

### Defining Disciplines and Strategies

A majority of the studies mentioned above used journal-based categories or higher level groupings of such categories, ranging from seven (King) and 56 (Chen) categories, to represent strategies that could be pursued by nations. However, use of journal-based categories to represent the structure and partitioning of research is problematic. Journal-based categories are disciplinary (they represent historical institutional structures) rather than cognitive (reflecting how scientists are self-organizing around problems). Collins [17] questioned the use of disciplinary categories for policy-making over 30 years ago when he wrote: “… to develop a policy with cognitive goals in view, it is essential to start by disaggregating science according to cognitive rather than institutional boundaries–that is, to think of science as being made up of sets of research areas which involve scientists who interact, or mutually refer, across institutional boundaries, because of their common cognitive interests.” This perspective is reflected in the current emphasis on interdisciplinary and translational research. [18]

**Fig 1:**
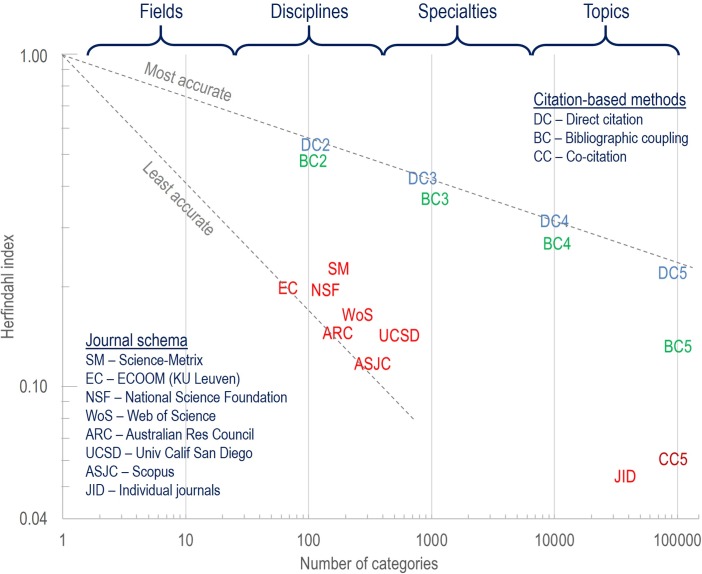
Relative accuracies of 17 document clustering solutions, adapted from reference 19. Designations of fields, disciplines, specialties and topics in terms of numbers of categories across all of science are not meant to be exact, but rather conceptual.

The problematic nature of journal-based categories became apparent in a recent study where the accuracy of different document partitioning strategies was compared. Using Scopus data, Klavans & Boyack [19] compared seven different journal classification systems with article-level classifications at different levels of granularity created using direct citation, bibliographic coupling, and co-citation methods (see Fig 1). Comparisons were made using a Herfindahl (or concentration) index calculated using the references in over 37,000 papers with at least 100 references each. These articles with large numbers of references were considered ‘gold standards’ because they tend to provide a comprehensive review or synthesis of a topic area. The Herfindahl index measures how closely a classification system preserves the topic structures as elucidated by the authors of the ‘gold standard’ articles. A high concentration value signifies that the classification system is structured in a way that corresponds with the authors’ perception of topical structure, while a low concentration value suggests that the classification system does not correspond with the authors’ logic. Comparing the accuracy of classification systems with different numbers of clusters can be done by comparing slopes of lines from the (1,1) point to the different Herfindahl values; the method with the least negative slope is the most accurate.

Fig 1 makes a distinction between classification systems at different levels, from disciplines (hundreds of categories, as in most journal classification systems) to topics (tens of thousands of categories, similar to Kuhnian research communities). Most relevant to this study, it shows that at the discipline level article-based classification systems (DC2 and BC2) do a much better job than any of the journal classification systems at reproducing structures defined by authors, and thus can be considered to be more accurate from this point of view. Article-based classifications also have the advantage that they reflect the cognitive structure of science, and thus satisfy Collins’ argument far better than do journal-based analyses. In addition, we have recently shown that publication profiles of most institutions are more aligned with the DC2 classification system than with journal classification systems [20]. Thus, in this study we will use the 114 DC2 discipline-level categories for our analysis. These were defined by clustering roughly 50 million documents from the Scopus database using direct citation. A description of these 114 disciplines is available in S1 Table.

In summary, there has been significant progress in the ability to identify national research strategies. King and May started with literature datasets that were somewhat limited in scope. Broadly defined disciplines were used to detect national research strategies. More sophisticated methods to normalize, and to detect the underlying dimensionality of choice, have been applied. Most importantly, a more accurate method for identifying cognitive-based structures in the literature is now available. These improvements provide the foundation from which our methodology will be built.

### Motives for Research

Historically, the primary justification for investing billions in research has been based in economics. When Carl Linnaeus (1707–1778) asked the King and Queen of Sweden to support his efforts at creating plant taxonomies, he argued that, if successful, he would be able to create cold-hardy plants that could be grown in Sweden, thereby allowing Sweden to develop national wealth based on agriculture [21]. In the 19th century, the U.S. government succeeded in making agriculture the basis for national wealth via the establishment of regional agricultural colleges and agricultural extension programs. In the 19th and early 20th century, different nations invested in basic research that supported national advantages in applied research (Germany in chemistry, France with its polytechnic schools). The industrial strength of the U.S. at the turn of the 20th century was due to entrepreneurs such as Carnegie, Rockefeller and JP Morgan, who exploited the link between science and invention in a nation that had few restraints on capitalism. The 1930’s saw the rise of large industrial laboratories as the source of innovation and economic growth. The 1970’s marked the decline of these large labs, a shift to open innovation systems and the resurgence of Europe and Japan as research leaders. In the past 10 years, China’s scientific and technical publication activity has risen from 24% of the U.S. output (in numbers of articles) to 97% of the U.S. output [22], with a corresponding rise in economic power. Overall, there is an extensive literature on the relationship between science, invention, innovation and economic growth that, in essence, points to economic advantage as the primary motive for research [23–27].

Why then, in this context, would the United States spend eight billion dollars to develop and launch the James Webb Space Telescope (JWST)? Although spillover benefit is often used to justify investments in astronomy, space exploration, and other basic sciences [25], the primary reason these things are funded is to gain knowledge, to seek answers to big problems. For instance, the JWST isn’t designed to seek out economic opportunities; it’s specifically designed to answer basic questions about the birth of the universe. Likewise, why do researchers choose careers in basic sciences rather than those that directly benefit the economy? Interviews with researchers in astronomy and space exploration reveal part of the answer. Astronomers are, in large measure, men and women who, as children, would look up at the stars in awe. They maintained this sense of awe in choosing majors and career paths. Personal motives for going into astronomy and astrophysics are not based on homo-economicus; they are based on curiosity–an altruistic analog. At both the researcher and funding levels, much of research is being done for reasons that are clearly not economic in nature. A different part of the national brain is being stimulated when altruistic research is considered.

We suggest that altruism is a dominant motive for research. Discovering basic principles of life and nature and making them manifest are an integral part of the human psyche. These research activities, along with those aimed at reducing illness, protecting the weak, providing world-wide educational opportunities, sustaining the earth as our home, reflecting on the meaning of life and living in a fair and just civilized state, are rooted in altruism. It is this tension–between our current belief about the role of research in economic progress and our observation that many research activities are strongly rooted in altruistic motives–that is the focus of this background discussion. As such, we will first elaborate on the traditional views of research and economic growth before we turn our attention to the possibility that altruism plays a significant role in why nations spend tens of billions of dollars on research. The background section concludes with a summary of how one can investigate these issues using publication data.

### Economic Competition and Research

Joseph Schumpeter (1883–1950) significantly influenced the economic policy in the United States and Europe via his focus on entrepreneurship, technological innovation and corresponding waves of economic destruction [28]. His position countered the Marxist’ criticism of capitalism at the turn of the 20th century. And while his beliefs about the advantages of capitalism changed when the large industrial labs were being formed in the 1930’s (i.e., due to a realization that socialism and capitalism are equally likely to benefit from investment in R&D), the underlying belief that there are long-term cycles in research and economic growth still holds merit [29]. Modern day economic theories about subsidizing R&D [30, 31] and the expected growth that will result from these investments are deeply rooted in Schumpeterian theory. Since patents are a primary mechanism for capturing the benefits from research, there is a corresponding literature on what subjects are patentable [32, 33]. Overall, there is a significantly large literature in the economic discipline that assumes that R&D is, in essence, motivated by economic considerations.

The field of strategic management also focuses on economic motives for research. Early work on corporate diversification, growth and profitability pointed out that the ‘exploitation of a scientific base’ was one of the three avenues for successful diversification [34]. This stream of research on corporate diversification was re-interpreted using the concept of absorptive capacity [35]. Absorptive capacity is the ability of a firm to identify, assimilate and apply external knowledge. This is now the dominant explanation for corporate research; firms conduct research in order to learn about what moves to make in the competitive environment and whether first mover advantages exist [36]. From the perspective of strategic management, corporations do not publish scientific articles for the altruistic reasons of contributing to world-wide knowledge and well-being.

Both economists and managers agree that entrepreneurs play a critical role in converting research into economic benefits. As such, there has been an extensive literature on the role of entrepreneurs, especially in regional growth. Starting with Roberts’ study of technical entrepreneurs from MIT that formed the Route 128 firms around Boston [37], there are now many studies that look at academic entrepreneurs [38], how the entrepreneurs at universities contribute to regional growth [39] and how incubators can be created (with corresponding research subsidies) in order to foster regional innovation [40]. There is also an extensive literature on the characteristics of these entrepreneurs and how entrepreneurs recognize opportunities [41–43].

The practical problem of taking research to the marketplace has also been addressed by academics studying the innovation process. Most notable were the early work on the characteristics of successful vs. unsuccessful new products [44] and the diffusion pattern of innovation [45]. As the source of innovation shifted from the large industrial labs, there has been corresponding work showing how innovation could be financed using crowd sourcing [46].

In summary, the majority of the academic literature that addresses the question ‘why do we do research?’ is deeply embedded in the assumption that research has economic benefits. Joseph Schumpeter is one of the most influential forefathers of this research, and there are many well developed research questions that are currently being investigated from the underlying assumption that inventors convert ideas into patents and entrepreneurs convert opportunities into product and process innovations. The assumed goal is economic gain or is strongly related to economic gain, such as is the case for research that enhances national security.

### Altruism and Research

Altruism provides a strong alternative to the economic motive for research. For example, the economic motive is not the dominant reason behind funding for the JWST (as mentioned above), nor is it the driver for the large amounts of funding that are directed (primarily by wealthy nations) to detect and respond to pandemics that, while they generate fear, are relatively unlikely to significantly affect those nations. At the personal level, many medical researchers chose their professions in response to a desire to find a cure for a particular disease that affected a family member or friend. In addition, many non-profit organizations (NPOs), both large and small, fund research related to their causes and missions, all of which have a strong altruistic component. Altruism plays a significant role in research.

Despite this role, the relationship between altruism and research has not been subject to the same level of study as has the economic motive. Altruism has had no intellectual champion such as Schumpeter. While the economic motive for research has been the subject of thousands of articles in dozens of research communities, a sampling of which is mentioned above, the literature on altruism is focused on topics other than its relationship with R&D. Most altruism research is performed within the context of topics on biological fitness and behavior, cooperation (in the evolutionary dynamics and game theory sense), organ donation, professionalism (particularly in medicine), medical education, volunteerism, citizenship, and monetary donation. These are all important topics, but none of them address the relationship between altruism and R&D.

**Fig 2:**
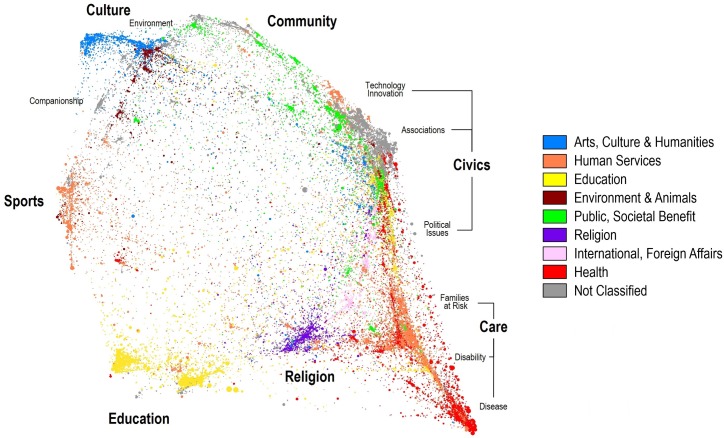
A map of altruistic missions for 125,000 NPOs in the United States. Each NPO is represented by a dot, and is color coded using high-level National Taxonomy of Exempt Entities (NTEE) categories (see legend).

In order to better understand the diversity of altruistic motives in non-profit organizations, we recently developed a ‘map of altruism’ based on a textual analysis of the websites of over 125,000 NPOs in the U.S. [47]. The relative positions of these institutions are shown in Fig 2, where each dot represents a specific NPO. Neighboring NPOs in map space share similar mission statements as reflected by the words used on their websites. Each NPO is color coded using the National Taxonomy of Exempt Entities (NTEE) system used by the Internal Revenue Service to classify NPOs.

Not surprisingly, one of the most populated areas on this map of altruism deals with the treatment of disease (lower right). NPOs in this area actively fund medical research without any expectation of economic gain. Their motives harken back to the same motives that were mentioned in the introduction–to extend life [1].

A comparable level of activity is associated with Civics. This area of the altruism map includes NPOs concerned about the type of society we live in and social dilemmas, as well as others that focus more on political issues. It is interesting to note that within Civics we see a large number of NPOs devoted to the issue of technological innovation. Consider, for example, the number of non-profits and regional development organizations that have been formed to encourage entrepreneurial behavior. While economists might argue that the motive of the entrepreneur (and the venture capitalist funding the entrepreneur) is personal gain, the NPOs helping entrepreneurs must (by law) be more altruistic.

NPOs interested in environmental issues are well represented in Fig 2 (top left) and have their own NTEE code. Many of these NPOs fund research on climate change and are concerned with the world’s ability to produce food for billions of people in the face of climate uncertainty. Related research on food production is part of the Agriculture strategy highlighted in Table 1.

Interestingly, many of the topics of altruism research–specifically organ donation, medical education, citizenship, volunteerism, and monetary donation–underpin the areas of altruism shown in Fig 2, and reflect the variety of ways that people and institutions give of their time and resources to altruistic causes. This diversity of mission space is reflected in the scientific literature on altruism. To the extent that NPOs fund scientific research related to these missions, there is an implicit link establishing altruism as a motive for scientific research. However, this relationship as a motive for research has not been investigated in a scientific way.

Some might be tempted to equate the conception of altruism-driven research with so-called Mode 1 research [26], which is defined as research motivated by curiosity or the desire for knowledge. However, we note that research driven by altruism can be application-oriented just as easily as it can be knowledge-oriented. In fact, the mission space of Fig 2 provides ample evidence that many, if not most, altruistic activities are aimed at making some sort of positive change rather than simply learning something new. Overall, we suggest that altruism is a strong motivator for R&D, despite the fact that the economic motive dominates the current research agenda. There are no indicators that tell us if an area of research is driven by altruistic or economic motives. There are no studies that utilize publication profiles of thousands of institutions from around the world to characterize fields of research. Finally, there are few studies that systematically explore a number of alternative reasons for a nation to focus on one field or another. The first two gaps are methodological, while the third is theoretical and is correspondingly addressed in the following sections.

## Methods

Very little work has been done to explore why institutions or nations might focus on altruistic research. Our high-level working hypotheses are that 1) some disciplines and strategies will be clearly motivated by economics while others will be largely motivated by altruism, and 2) that some combination of economic, social, cultural, or political indicators will explain differences in national research strategies.

**Fig 3:**
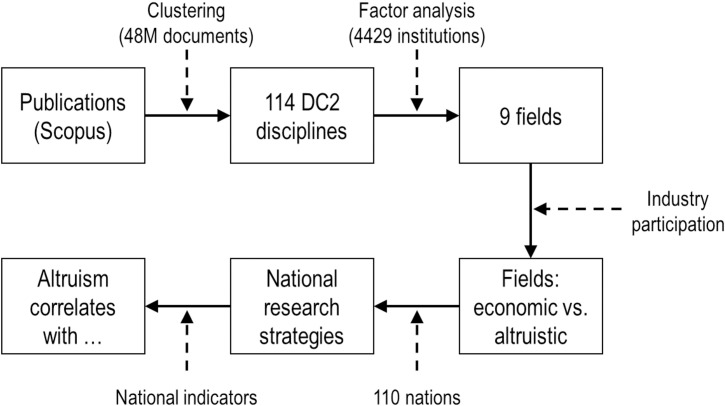
Schematic of study methodology.

Fig 3 summarizes the methodological steps used to explore these issues. The three boxes at the top of Fig 3 focus on how we have improved the way that a literature database is structured. The clustering step was performed previously [19], resulting in the 114 disciplines which, as pointed out in Fig 1, are a more accurate characterization of the cognitive structure of research than any journal-based disciplinary schema. We then used factor analysis to reduce the dimensionality of the data from 114 disciplines to nine fields. Unlike the studies summarized in Table 1, however, we used the publication profiles of 4429 institutions instead of the publication profiles of the 110 nations to identify these fields.

The use of 4429 institutional publication profiles enables more accurate identification of the underlying structure in the data than would use of 110 national publication profiles. Doré [11] identified a factor (a group of disciplines and countries) associated with agriculture, and confirmed this interpretation by qualitative means–i.e., looking at the labels of the disciplines and the names of the very few nations that loaded most highly on this factor. A much clearer signal is obtained using institutions. Using the agriculture example, there are dozens of agricultural colleges and agricultural research institutions around the world whose publication profiles tell us what disciplines should (or should not) be associated with agriculture. If these institutions load highly on a single factor with high coefficients, one can be certain that the factor is about agriculture. In cases that are less clear, one can refer to institutional websites to identify their strategies and use that evidence to characterize a research strategy.

The three boxes at the bottom of Fig 3 focus on our working hypotheses. We have used the level of industry participation to determine if a discipline or field is more aligned with altruism or economic motivation. High industry participation reflects an economic motive for research, while low industry participation reflects a more altruistic motivation. We then generated national publication profiles using these nine fields. As a final step, we correlated the national research strategy profiles with national indicators of wealth, education, capitalism, culture, religion and language to determine which of these national indicators are associated with altruistic or economic motivations.

## Data

This study uses publication data from the Scopus database. Publication profiles for institutions and nations are restricted to the 2010–2013 time period.

### Institutions

We identified a set of 4429 institutions for use in this study. Institutions were identified manually several years ago by grouping Scopus affiliation profiles into institutions for those institutions publishing at least 50 papers per year (at the time). Many institutions have only a single affiliation profile in Scopus. However, many of the larger institutions have medical (or other) schools or research institutes that, while they are clearly part of the parent institution, have separate affiliation profiles in Scopus. For example, our institutional profile for the University of California, San Diego (UCSD) contains six affiliation profiles including those for the Scripps Institution of Oceanography, UCSD School of Medicine, and San Diego Supercomputer Center. Each institution was also assigned to a sector (e.g., academia, industry, government), which was, in most cases, an obvious choice. Given that these institutional groupings are several years old, they do not reflect recent mergers or contain institutions that were below the threshold at the time, but that are now publishing larger numbers of papers. The distribution of institutions by sector is shown in Table 2. On average, academic institutions publish larger numbers of papers than other types of institutions.

**Table 2:** Institutions by sector (2010–2013).

Sector	# of Institutions	Avg # of Articles
Academia	2603 (58.8%)	4219
Government	711 (16.1%)	1590
Hospitals	553 (12.5%)	767
Industry	291 (6.6%)	847
Non-profit	142 (3.2%)	1383
Academy of Science	121 (2.7%)	2325
Other	8 (0.2%)	1932
Total	4429	2998

As mentioned above, the DC2 document classification system is used in this study. Thus, publication profiles for each institution were represented as article counts by DC2 category using publications from the 2010–2013 time period. These data also enable calculation of the fraction of publications in each DC2 that are authored by industrial institutions, which indicates whether a DC2 discipline is aligned more with economic motives (a relatively high industrial fraction) or with altruistic motives (a relatively low industrial fraction). We use this fraction as an indicator of motive later in the study.

### National Characteristics

**Fig 4:**
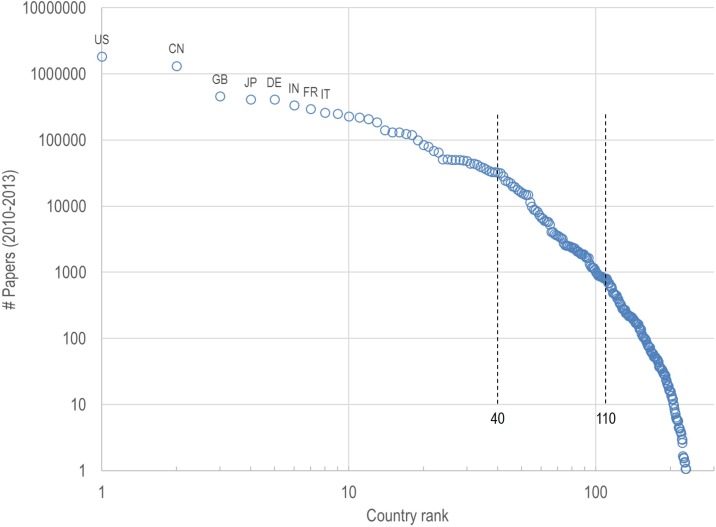
Distribution of national publication counts.

Publications in the Scopus database are assigned to 240 different countries. National publication counts range from over 1.8 million (USA) to less than a single paper (nine nations, e.g., Tuvalu) over the 2010–2013 time period using fractional counting and excluding any publication which could not be assigned to a DC2 discipline. The distribution of national publication counts in Fig 4 shows different regimes. For example, the distribution is relatively linear on a log-log scale for the first 40 nations, and is also relatively linear for the next 70 nations but with a different slope. Beyond the first 110 nations, the dropoff in publication rate is more severe. As such, we will limit our analysis to the ‘top 40’ nations (where one can expect a more intensive infrastructure for research) and the ‘next 70’ nations (with a relatively weak research infrastructure).

As mentioned above, one of the goals of this research is to correlate national research profiles with national indicators of wealth, education, capitalism, culture, religion and language to determine which of these other factors are associated with altruistic motivation at the national level. As such, we include a number of national level indicators in this study. The specific indicators used and their sources are listed in Table 3.

**Table 3:** National indicators and sources.

Category	Indicator and Source
Wealth	2015 **GDP per person**, International Monetary Fund version(https://en.wikipedia.org/wiki/List_of_countries_by_GDP_(PPP)_per_capita)
Education	United Nations **Education Index**, 2013(http://hdr.undp.org/en/content/education-index)
Capitalism	Frazer Institute **Economic Freedom Index**, 2013(https://en.wikipedia.org/wiki/Economic_Freedom_of_the_World)
Culture	Hofstede Center **Individualism Index** and **Power Distance Index**(https://geert-hofstede.com/countries.html)
Religion	**% Christian**(https://en.wikipedia.org/wiki/Religions_by_country)
Language	**% English** speaking(https://en.wikipedia.org/wiki/List_of_countries_by_English-speaking_population)

A description of these indicators along with the correlations we expect to see are given here.

- *Wealth*: Per capita GDP is a commonly used indicator of national wealth. We expect that only wealthy nations will fund high levels of research in altruistic disciplines and fields.
- *Education*: The United Nations’ Education Index is based on expected and actual years of schooling for each nation. Given the current belief that economies are largely knowledge-based [48], we expect that altruistic research will be associated with high levels of education at the national level.
- *Capitalism*: The Frazer Institute’s Economic Freedom Index represents a strong libertarian focus on issues such as personal choice (instead of collective choice), voluntary markets, freedom to enter and compete in markets and protection of property rights. This index also represents Schumpeter’s initial premise that R&D is more likely to flourish in capitalistic systems. We have no working hypothesis about which form of government is more likely to support altruistic research.
- *Culture*: We used two indexes created by the Hofstede Center related to culture. The Power Distance Index expresses the extent to which the less powerful members of society accept and expect that power is distributed unequally, while the Individualism Index reflects the degree to which people believe that they should take care of themselves or prefer being in a tight-knit group in exchange for loyalty to that group. We have no working hypothesis about whether the cultural aspects of a nation are more or less likely to be associated with altruism.
- *Religion*: We include an indicator of religion simply because religion is clearly an important element in altruistic motivations, as indicated by our map in Fig 2, and because religion has played a historical role in how science has evolved over centuries. We use percent Christian as the indicator since it is the dominant religion in 73 of the 110 nations included in the study, although data on other religions (Muslim, Buddhist, Hindu, Jewish, etc.) are also available from the original source.
- *Language*: Degree of English-speaking is used as our indicator of language. This indicator is included because the Scopus database used to generate publication profiles focuses mostly on English publications. We note, for example, that there are extensive literatures on Chinese medicine and Ayurvedic medicine that are not well covered in Scopus (or other major databases that cater to the Western world). Many engineering conferences and other conferences of a more applied nature are published in local languages and may be under-represented. Our working hypothesis is that English-speaking nations are more likely to be associated with altruism, but that this may reflect database bias.

Indicator values for the 110 nations in our sample are provided in S1 Table. Complete data (for all 110 nations) were available for three of the indicators: wealth, religion and language (see Table 4). The education indicator had 4 missing values while the culture indicators are missing for 24 nations. Values for language are missing for 38 nations; we have used estimated values of 1% English-speaking for the 4 missing values among the top 40 nations, but have not used estimates for the other 70 nations.

**Table 4:** Characteristics of the nine research strategies identified using factor analysis.

	Civics	Med	Disease	Sustain	Biochem	B Phys	Comput	A Phys	Eng
Summary Statistics									
%Industry	2.92%	4.85%	6.08%	6.49%	6.65%	7.07%	8.19%	10.35%	14.28%
#DC2	13	27	7	12	12	5	11	11	16
#Doc (million)	1.398	1.857	0.316	0.935	0.927	0.268	1.269	1.028	0.925
Focus (%Docs)	15.7%	20.8%	3.5%	10.5%	10.4%	3.0%	14.2%	11.5%	10.4%
% Growth	7.1%	5.8%	6.1%	5.7%	5.0%	1.6%	3.2%	5.8%	6.8%
Nations/Paper	1.41	1.76	1.78	1.59	1.61	1.84	1.33	1.45	1.42

In summary, our hypotheses are mostly exploratory in nature. We expect that factor analysis will group disciplines into higher-level fields of research that are internally consistent and valid. We expect that the participation of industry in a field of research will create a reasonable ordering of those fields in terms of altruistic motivation. While researchers have suggested that wealth, education, capitalism, culture, religion and language will explain why nations focus on different strategies, we consider these as alternative hypotheses and will let the data tell us which explanations best explain variations in national focus along a potential altruism-economic axis.

## Results

As mentioned previously, our methodology is comprised of four main steps– 1) grouping disciplines into fields using factor analysis, 2) characterizing fields with respect to motive, 3) defining national research strategies using fields, and 4) correlating research strategies and motives with national indicators. Results of each of these steps are detailed in this section.

### Factor Analysis

Nine research fields were identified by grouping of DC2 disciplines using factor analysis. Inputs were the publication profiles of the 4429 institutions across the 114 disciplines. Oblique rotated factors were used to allow factors to be inter-correlated because, a priori, there is no reason to believe that fields of research are orthogonal [49, 50]. The factor analysis generated, as output, vectors that represent ‘underlying dimensions of choice’. In essence, disciplines that occur together across institutional publication profiles tend to load strongly together on the same factor, and are thus grouped in the same field.

The factor analysis produced seven factors with an eigenvalue of greater than 1.0. Two of these factors represented alternative (or opposed) fields, where there was one group of disciplines with highly positive coefficients and another group of disciplines with highly negative coefficients. For instance, one of the pairs of opposed fields is sustainability and applied physics; institutions are typically focused on one or the other, but not both. These two factors resulted in four fields. The remaining five factors each represented a single field rather than a pair of opposed fields in that positive coefficients were high and negative coefficients were near zero. In total, a set of nine fields were identified. Characteristics of the 114 DC2 disciplines and their assignments to the nine fields are given in S2 Table.

### Field Characterization

The nine research fields identified using factor analysis have been characterized in a number of ways. In this section we name and describe each field, and quantify each with respect to industry participation. Industrial authors were identified as those associated with one of the 291 industrial institutions from Table 2, or whose affiliation data as indexed in Scopus contained one of the industry-related strings given in S3 Table. The major source for these strings was Wikipedia (https://en.wikipedia.org/wiki/Types_of_business_entity), with some additional strings suggested by Sugimoto et al. [51]. We note that the string-based search was essential given that the results from our 291 (large) industrial institutions only identified 1/3 of the industry authorships compared to the full search strategy. This suggests that 2/3 of industry-authored papers are coming from smaller companies, those that publish less than 50 papers per year.

Following is a discussion of each field of research, starting with the label we have used for naming. Labels for each field were assigned manually and were based on examination of the DC2 disciplines and the institutions with the top coefficients for each field. Fields are ordered by increasing fraction of industry authorship. For purposes of discussion, we have defined two industry participation thresholds. Overall, industrial authors participated on 7.07% of papers from 2010–2013. We define disciplines with more than 10.0% industry authorship to be in the ‘economic’ range (26 disciplines, 21.6% of papers), while those with less than 4.0% industry authorship are in the ‘altruism’ range (23 disciplines, 20.5% of papers). Numbers in parentheses in the descriptions below refer to DC2 discipline numbers (see S2 Table).

#### 1—Civics

The first field of research includes 13 disciplines commonly found in a college devoted to a liberal arts education. Seven disciplines are below the threshold (very little industry involvement). Those disciplines with the least industry involvement include Medieval Studies (22), Governance (0), Philosophy (95) and Learning (14). The discipline with the most industry involvement is about Sound (90), and involves music, acoustics and speech. This field is clearly associated with the section of the altruism map (Fig 2) that is labeled ‘Civics’.

#### 2—Medicine

The second field of research has 27 disciplines associated with the treatment of patients, and is the largest of the nine fields. The more altruistic disciplines are Pregnancy & Childbirth (62), Emergency Medicine (71), Neurology (75) and Gastrointestinal Science (37). Those disciplines with higher than average industry involvement are Medical Imaging (106), Respiratory Diseases (55) and Tobacco-related illness (111).

#### 3—Infectious Disease

This field is relatively small and highly focused. The more altruistic disciplines focus on Tuberculosis (98), Tropical Diseases (42) and Mycology (107). The disciplines with the most industry involvement are Virology (78) and Veterinary Science (92). It is appropriate that Veterinary Science is located in this group in that many infectious diseases jump from animals to humans, and veterinarians are among the first professionals who detect these emerging threats.

#### 4—Sustainability

This field of research contains 12 disciplines that are linked because they deal with many issues commonly associated with sustainability, in terms of both science and policy. The most altruistic discipline is an extremely small discipline that focuses on Public Health Policy and other issues in Brazil (104). The large disciplines that are more altruistic include Marine Science (5), Ecology (21), Wildlife Science (30), Plant Science (3) and Entomology (31). The disciplines that are most associated with industry focus on Forestry (100) and the Geosciences (4). The disciplines that blend altruistic and economic motives include Climate Science (54) and Environmental Engineering (81).

#### 5—Biochemistry

The 12 disciplines in this field of research are all related to the chemistry of living creatures. The more altruistic research is on Medicinal Chemistry (36), Alcohol (97), Biochemistry (110) and Molecular Chemistry (1). Those disciplines that include more industrial firms include Pharmacology (68) and Analytical Chemistry (77).

#### 6—Basic Physics

This is the smallest research field, and focuses on only a few disciplines where there tends to be significant international collaboration among theoretical physicists. The discipline with the least industrial involvement deals with Particle Physics (32). This field is where one finds researchers studying Planetary Science (56), Astronomers interested in how the universe was formed (48), Nuclear Medicine (58) and Plasma Physics (94).

#### 7 –Computing Technology

The 11 disciplines in this field are primarily based on advances in computer science and related work in mathematics and applied physics. Labeling this group as ‘computer science’ would be misleading, because the disciplines are mostly aimed at application areas. New industries are forming around Computing (6), particularly cloud computing, Telecommunication (52), Networks (27), Human Computing (67), including the creation of virtual environments and smart homes, Cryptography (93) and Computer Vision (9). Mathematics (20) and Nonlinear Dynamics (102) are clearly more altruistic in nature, but are in this field because they provide the theoretical underpinnings for the more applied work.

#### 8—Applied Physics

This field groups disciplines dealing with materials and applications based on physics, many of which find their way into electronics and other industries. Lithography (113), Electronic Packaging (108), Semiconductor Physics (15), and Optoelectronics & Photonics (38) lead the list where industry is highly involved. Included in this group is work on Carbon Science (64), including graphene, and Nanochemistry (41).

#### 9—Engineering

All of the 16 disciplines associated with engineering have industry involvement that is higher than the average for the full database (>7.07%). The most economic-oriented disciplines deal with Petroleum Engineering (105), Power & Electricity (43), Nuclear Science (94) and Geological Engineering (53).

**Fig 5:**
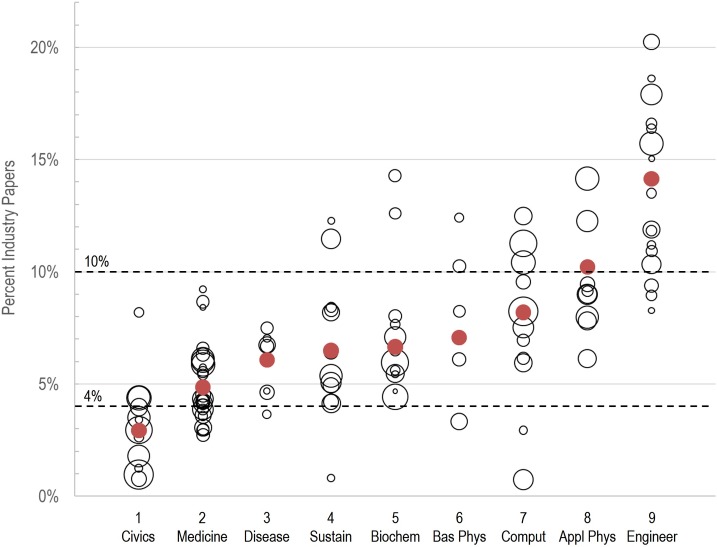
Industry involvement in 114 DC2 disciplines assigned to nine fields of research. Each discipline is represented by its number as listed in S2 Table. Circle sizes reflect discipline sizes while filled circles indicate average industry authorship by field.

Overall, the factor analytic approach has created a very coherent grouping of 114 disciplines into nine fields, characteristics of which are given in Table 4. Medicine is by far the largest field with nearly 21% of papers, while Infectious Disease and Basic Physics are both quite small, with around 3% of papers each. The rest of the fields are more similar in size. In addition, Fig 5 shows that not only do the nine fields reflect a continuum from less industry involvement (more altruistic) to more industry involvement (more economically motivated), but that there are few altruistic disciplines in economic fields, and few economic disciplines in altruistic fields, suggesting that some fields (and disciplines) are inherently more altruistically motivated while others are more economically motivated. Industry involvement provides a very reasonable ordering of fields and disciplines within each field (see S2 Table).

The field with the least involvement by industry is Civics. In addition to being the most altruistic field, Civics is the second largest and fastest growing field. However, it has a surprisingly low level of international collaboration; the average paper in this group had 1.41 national affiliations. Those fields with the highest industrial involvement are where one most feels the Schumpeterian winds of creative destruction, especially the fields of research based on Applied Physics and Engineering.

International collaboration patterns may indicate whether the field of research represents local or global issues. For example, Basic Physics, Medicine, and Sustainability exhibit relatively high international collaboration. Conversely, those fields where industry is most involved–Engineering, Computing Technology, and Applied Physics–have lower values. The lower level of international collaboration for the Civics field may indicate that this strategy is mostly dealing with local, rather than global governance issues.

### National Research Strategies

**Fig 6:**
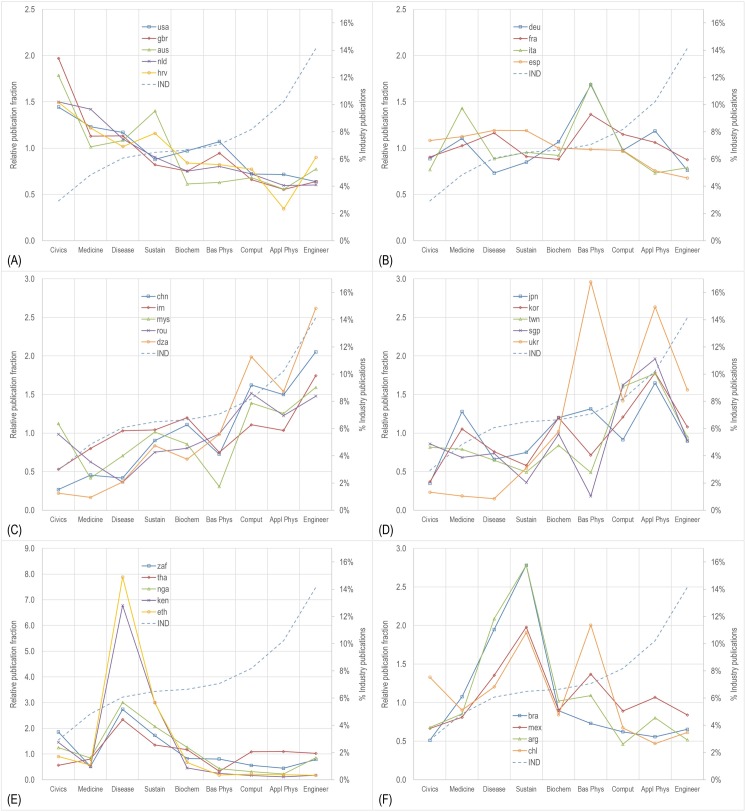
Relative publication fraction portfolios for six different groups of countries: a) civics portfolio, b) balanced portfolio, c) engineering portfolio, d) nanotech portfolio, e) disease portfolio, and f) sustainability portfolio.

Using the fields defined and characterized in the previous sections, we can now examine which nations focus on which fields of research. Fig 6 provides relative publication fractions by field for six different groups of nations, with each group having a different profile. Relative publication fraction is the fraction of documents by a nation in that field divided by the fraction of documents worldwide in that field (“Focus” in Table 4). For example, the US has 22.6% of its output in Civics, which is 1.44 times the world average of 15.7%. National publication fractions by field are given in S4 Table.

Fig 6A shows publication fractions for five countries (usa, gbr, aus, nld, hrv) whose portfolios by field are roughly opposite to the industry publication profile by field that is shown for comparison as the dashed line. These countries publish proportionally more in fields with relatively low industry participation, and proportionally less in fields with relatively high industry participation. One might say these countries have a Civics-oriented portfolio that is not consistent with overall industry (or economic) profile. Fig 6B shows a much more evenly distributed set of portfolios belonging to several European countries (deu, fra, ita, esp), with the exception of the spike in Basic Physics for Germany and Italy (and France to a lesser degree) that is due to their involvement in CERN.

Fig 6C and 6D show two different types of portfolios that both roughly follow the industry participation (or economic) profile. They differ in that Engineering predominates for some countries (Fig 6C: chn, irn, mys, rou, dza), while Applied Physics or materials and nanotechnology dominates for others (Fig 6D). There are two subtypes among the Applied Physics dominant countries, those that also focus on Basic Physics (jpn, ukr) and those that focus on Computing applications (kor, twn, sgp). The final two panels show countries whose portfolios are focused on Infectious Disease (Fig 6E: zaf, tha, nga, ken, eth) and Sustainability (Fig 6F: bra, mex, arg, chl), respectively. Chile also shows a spike in Basic Physics, which reflects its relative strength in Astronomy associated with their world-class observatories.

These six different types of portfolios show a great diversity in national research focus, and suggest that motives other than economic benefit, such as health, quality of life, and environmental concern, are drivers in many nations. Although some of the groupings in these examples seem to fall along geographical lines, there are also many cases where geographies are mixed in non-intuitive ways. For example, Iran, Romania, Algeria (irn, rou, dza) have Engineering-dominant profiles similar to that of China, and Croatia (hrv) has a Civics-dominant profile like that of the USA.

**Fig 7:**
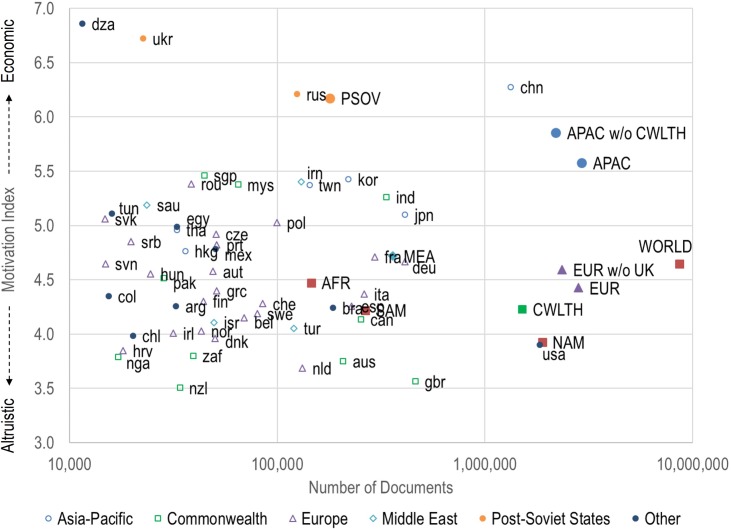
Motivation index for the 54 nations with at least 10,000 publications from 2010–2013. Index values for various aggregations of nations are also shown for comparison.

To give an overall idea of whether the research agenda of a nation is more aligned with an economic or altruistic profile, and for the purposes of correlating national research profiles with other characteristics, we have reduced motivation to a single value. We propose a motivation index based on the distribution of a nation’s publications over the nine fields. The motivation index is calculated for a nation as M = ∑ f p_f_, where f is the field number (1–9) and p_f_ is the fraction of papers in that field for that nation. Fractions are listed for each nation in S4 Table, along with the calculated value of the motivation index. Fig 7 shows these index values as a function of number of publications for all nations with at least 10,000 papers published (summed fractional counts) from 2010–2013. It also shows that the motivation index for full set of 110 nations in this study is 4.65. The fact that this index value is less than 5.0 reflects the fact that there are more publications in the more altruistic fields (1–4) than in more economic fields (6–9).

The two top-producing nations provide a sharp example of the difference between a more altruistic research strategy (USA, M = 3.90) and a more economic research strategy (China, chn, M = 6.28). The USA has a majority of its publications in Civics and Medicine, while China focuses on Engineering, Computing and Applied Physics. In contrast, many European nations, including Germany and France (deu, M = 4.67; fra, M = 4.71), have a more balanced research portfolios with motivation index values very close to the world average. In general, northern European nations (e.g., nld, dnk, nor, swe) have a slightly more altruistic focus than the rest of the European nations.

Fig 7 also shows the motivation index for a number of continents and two additional aggregations of countries–Commonwealth countries and Post-Soviet States (assignments are given in S1 Table). Several of these aggregations are simple reflections of their dominant country–e.g., North America and the US, South America and Brazil, and the Post-Soviet States and Russia. The Middle East and EU (and more particularly the EU without the UK) have the most balanced strategies. The Commonwealth, consisting mostly of former British territories, is comprised of two components–one which is less economically motivated (i.e., aus, can, nzl, zaf, nga) and mirrors the UK (gbr), and one which is more economically motivated (i.e., ind, mys, sgp) and reflects the Asia-Pacific philosophy. In the latter case, geography seems to matter more than the former British influence. Possible reasons for national differences are examined in the following analyses.

### Field-Indicator Correlations

The final step in our analysis was to correlate national indicators with the motivation index to determine if any of the indicators might suggest reasons for differences between altruistic and economic motivations for research. Table 5 shows that the values of these correlations for the top 40 nations and the next 70 nations (those ranked 41–110) are significantly different. For the top 40 nations, the two most explanatory national indicators are individualism and religion. Although power, language and education have higher correlations with the motivation index than does religion, they are highly correlated with individualism (>0.63 in each case), and are thus less explanatory than religion, which has much smaller correlations with these variables. The next 70 nations have much weaker correlations overall, with individualism having only the fifth highest correlation.

**Table 5:** Correlations between motivation index and national indicators.

	Top 40	Next 70
Individualism	-0.742	0.186
Power	0.707	0.216
Language (% English)	-0.645	-0.290
Education	0.494	0.346
Religion (% Christian)	-0.414	-0.306
Wealth (GDP)	-0.276	0.160
Capitalism (Econ Free)	-0.241	-0.001

A common perception is that only the most developed nations can afford expensive research or research that is not directly aimed at economic gain. Indeed, we expected to find that altruistic research would be associated with high levels of wealth.–However, this was not substantiated by the data. The correlations between wealth and motivation are among the lowest for both sets of nations. Moreover, Fig 6 shows a number of poorer nations that have research portfolios that are similar to more wealthy nations, and also shows that nations with particular needs in Disease and Sustainability focus much of their research in those areas rather than pursuing research in more economically oriented fields.

We also expected that motivation would correlate strongly with education. It is more strongly correlated than is wealth, but it is also highly correlated with individualism, which is the more explanation feature. We did not expect any correlation between motivation and capitalism, and indeed there is very little.

**Fig 8:**
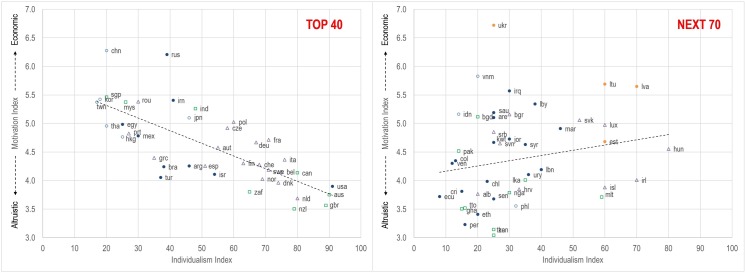
Motivation index as a function of individualism for the top 40 nations and next 70 nations. Legend as in Fig 7.

Fig 8 visually shows the relationships between our motivation index and the individualism index for the top 40 and next 70 nations. The correlation between individualism and motivation for the top 40 nations is strong and negative (Fig 8, left); nations with a high individualism index are likely to have research portfolios that align more strongly with altruistic motives, while those with low individualism indexes are likely to be more economically motivated. This correlation flips, however, for the next 70 nations–the correlation is positive rather than negative, and there is also much more variance in the distribution (Fig 8, right). The two distributions and corresponding correlations are markedly different, suggesting that our choice to split the nations into two regimes (top 40 and next 70) was reasonable. We stress here that these are correlations only, and do not indicate causality. In addition, it is clear that none of the national indicators that correlate highly with the motivation index are independent, and thus the indicators are suggestive rather than explanatory.

## Discussion

The objectives and results of this study differ from those in previous studies in several ways. First, and perhaps most importantly, this work introduces the role of altruism in national research strategies into the question–it has not been addressed before–and has shown that while some disciplines, fields of research and nations are more aligned with economic motivations, others are aligned with other motives such as societal good, health and quality of life, which we group together as altruistic motives. We note that there may be an objection to our use of the concept of ‘altruism’ as being opposed to economic motivation. In both formal and informal presentations and discussions with colleagues we have encountered those who resonate with the idea of altruistic motives and those who feel we use the term too broadly. Others may choose to use a different descriptor. Our choice reflects our previous work [47] and continuing interest in altruism along with the fact that we are continually exposed to researchers who pursue their research simply because they feel it will benefit mankind–because they want to make a difference in the world.

Second, our analysis suggests that research can be grouped into nine major ‘fields’ that are different in scope than those posited as strategies by previous studies. For example, the strategies listed in Table 1 fall into three major categories–Basic S&E (or Natural), Biomedical (or Life, Medicine), and Agricultural (or Developing). Eight of our nine fields can be mapped to these three major categories (see Table 6). However, our Civics field cannot be mapped to any previous categorization. Most of the previous studies excluded data from the Social Sciences, so they cannot be expected to have identified this field. Nevertheless, this field is extremely important from an altruistic point of view since it has the lowest industry penetration, and is a defining part of strategy for many nations, notable the USA and many Commonwealth nations. We note also that the ordering of our nine fields is reasonably consistent with the three major strategies identified by previous studies. Only Sustainability is out of order when compared with previous high level characterizations of national research strategies.

**Table 6:** Mapping of nine fields from this study to the three major strategies obtained by the majority of studies in Table 1.

This study	Previous studies
1. Civics	N/A
2. Medicine	Biomedicine
3. Infectious Disease	
5. Biochemistry	
4. Sustainability	Agriculture
6. Basic Physics	Basic S&E
7. Computing	
8. Applied Physics	
9. Engineering	

We note once again that the USA and China, the two countries with the highest scientific production, are exemplars of more altruistic and more economic national research strategies, respectively, as shown in Fig 6. On the one hand, the USA, with a strong sense of individualism and a tendency to question authority, has a focus that is tilted toward Civics and Medical Research. China represents a strategy at the other end of the spectrum. It has a strong collectivist culture and its research is tilted toward the three fields of research most associated with economic motives. There are natural questions that arise from examination of the differences in national cultures and strategies. For instance, if both countries continue to pursue their current scientific strategies, will the economic gap between countries decrease more quickly?

Finally, we have shown that the continuum from altruistic motivation to economic motivation correlates strongly with a number of national indicators, notably individualism and religion, both of which were surprising findings. National wealth is not strongly correlated with national research strategy. Although this finding contradicts popular wisdom, it does not necessarily contradict the pioneering work of May and King because they studied impact rather than strategy.

There are a number of potential weaknesses in this study that should be pointed out. First, we must point out that any publication database that one uses is biased. Early studies using the ISI databases (i.e., Science Citation Index) had the problem of being biased towards English and against the more applied literature. Both the Web of Science and Scopus databases have dealt with these issues to some degree by expanding coverage and, particularly, adding conference papers. However, biases still exist toward English-language journals, and coverage in the Social Sciences and Humanities are still at a large deficit related to coverage in the Natural and Medical Sciences. These biases obviously color the results of any study of national strategy because each nation is affected by bias in a different way. Given the relative lack of coverage in the Social Science, it is possible that altruistic activity in science is still under-represented.

A second potential weakness is our use of industry publication fractions as a proxy for economic motivation. We are well aware that firms tend to patent rather than publish, and that patents are typically preferred over publications as the basis for economic indicators. Linking patents to our field structure would provide a means to perhaps create a more direct linkage between industry involvement and economic motive. Nevertheless, we feel that industry publication is a strong indicator of economic motive. Since firms tend to patent rather than publish, they are only likely to publish in areas where they can economically benefit, and in that sense publications may be a very discriminatory indicator of economic motive.

A third weakness is that bibliometric studies are often difficult to replicate. Most studies of national strengths and focus have used journal classification systems (such as WoS subject categories) that are commonly available. However, in this study we introduce a new classification system (114 DC2 disciplines grouped into 9 fields) based on the full Scopus database. We acknowledge that replication of this structure will be difficult due to lack of data access. Nevertheless, exact replication is not necessary. For the past several years the Web of Science and Scopus have had similar coverage; thus, either database can be used for such a study. In addition, an open source algorithm [52] was used to create the DC2 disciplines, and the factor analysis method used to group disciplines into fields is commonly available. It has been shown that the structure of science is relatively robust at the level of 15–20 major partitions. [53] Given this robustness and the availability of clustering and analysis tools, we fully expect that anyone using a direct citation approach to cluster an entire citation database will get results that are conceptually similar to those presented here.

A final weakness may be in the indicators chosen to characterize nations. We used a broader (and perhaps more controversial) set of indicators than have been used in the past, and which some may argue are driven by ideology. In anticipation of potential ideological criticisms, we simply point out that we are quite willing to entertain other possibilities. We challenge researchers to find an alternative explanation using as much (or even more) data than have been used in this study. Despite these potential shortcomings, the overall picture presented by this study is both consistent with previous work, and expands upon it in a new direction.

We suggest that non-economic motives should be explicitly considered when addressing national research strategy. It is clear that a variety of national portfolio types exist (see Fig 6) and there is no reason to believe that any particular portfolio type is inherently any better than any other. This variety suggests that each nation pursues research to address its own set of needs, and that these needs reflect a variety of motives, only one of which is the economic motive. We note that the majority of science metrics are based on citations, and that these metrics are inherently more aligned with economic than non-economic motives in that they count scientific impact. Thus, most current indicators do a poor job of describing the returns from research whose motives may be social good, quality of life, environmental concerns, or any number of other altruistic activities. We urge policy and decision makers to consider that valuable outcomes result from more altruistically oriented research, and that metrics need to be devised to capture this properly. Finally, we call upon the research community to consider how we can measure outcomes from science driven by non-economic motives in a way that adequately communicates its benefits.

## Supporting Information

S1 Table National indicator values. (DOCX)

S2 Table Description of 114 DC2 disciplines and grouping into fields. (DOCX)

S3 Table Industry-related strings used to find industry authored papers along with the numbers of matches for 2010–2013 using institution strings and associated Scopus affiliation profiles. Due to overlap in the matches, the unique set of matches is less than the sum over strings and match types.
(DOCX)

S4 Table National publication fractions by field. (DOCX)
