# Supplementary material for: The Research Focus of Nations: Economic vs. Altruistic Motivations
Source: PLoS One. 2017 Jan 5;12(1):e0169383. doi: 10.1371/journal.pone.0169383 (PMC5215941; doi:10.1371/journal.pone.0169383)
Supplement: S1 Table — (DOCX) [file pone.0169383.s001.docx]

**Table S1. National indicator values.**

| **Rk** | **Country** |  | **Reg1 ^a^** | **Reg2** | **Wealth**  (GDPpp) | **Educ**  (Index) | **Capital**  (EcFree Index) | **Culture**  (Indiv Index) | **Culture**  (Power Index) | **Religion**  (%Chr) | **Lang**  (%Eng) |
| --- | --- | --- | --- | --- | --- | --- | --- | --- | --- | --- | --- |
| 1 | United States | usa | NAM |  | 55805 | 0.89 | 7.73 | 91 | 40 | 78.3 | 94.2 |
| 2 | China | chn | APAC |  | 14107 | 0.61 | 6.44 | 20 | 80 | 5.1 | 0.8 |
| 3 | United Kingdom | gbr | EUR | CWLTH | 41159 | 0.86 | 7.87 | 89 | 35 | 71 | 97.74 |
| 4 | Japan | jpn | APAC |  | 38054 | 0.808 | 7.52 | 46 | 54 | 1.6 | 1 ^c^ |
| 5 | Germany | deu | EUR |  | 46893 | 0.884 | 7.5 | 67 | 35 | 68.7 | 64 |
| 6 | India | ind | APAC | CWLTH | 6162 | 0.473 | 6.43 | 48 | 77 | 2.5 | 10.35 |
| 7 | France | fra | EUR |  | 41181 | 0.816 | 7.12 | 71 | 68 | 63 | 39 |
| 8 | Italy | ita | EUR |  | 35708 | 0.79 | 7.13 | 76 | 50 | 83.3 | 34 |
| 9 | Canada | can | NAM | CWLTH | 45553 | 0.85 | 7.89 | 80 | 39 | 69 | 85.63 |
| 10 | Spain | esp | EUR |  | 34819 | 0.794 | 7.27 | 51 | 57 | 78.6 | 22 |
| 11 | South Korea | kor | APAC |  | 36511 | 0.865 | 7.38 | 18 | 60 | 29.4 | 1 ^c^ |
| 12 | Australia | aus | APAC | CWLTH | 47389 | 0.927 | 7.83 | 90 | 36 | 67.3 | 97.03 |
| 13 | Brazil | bra | SAM |  | 15615 | 0.661 | 6.34 | 38 | 69 | 88.9 | 5 |
| 14 | Taiwan | twn | APAC |  | 46783 | 0.767 ^b^ | 7.76 | 17 | 58 | 5.5 | 1 ^c^ |
| 15 | Netherlands | nld | EUR |  | 49166 | 0.894 | 7.48 | 80 | 38 | 50.6 | 90 |
| 16 | Iran | irn | MEA |  | 17251 | 0.683 | 5.43 | 41 | 58 | 0.2 | 1 ^c^ |
| 17 | Russian Fed | rus | EUR | PSOV | 25411 | 0.78 | 6.69 | 39 | 93 | 73.3 | 5.48 |
| 18 | Turkey | tur | MEA |  | 20438 | 0.652 | 6.92 | 37 | 66 | 0.4 | 17 |
| 19 | Poland | pol | EUR |  | 26455 | 0.825 | 7.29 | 60 | 68 | 94.3 | 37 |
| 20 | Switzerland | che | EUR |  | 58551 | 0.844 | 8.16 | 68 | 34 | 81.3 | 61.28 |
| 21 | Sweden | swe | EUR |  | 47922 | 0.83 | 7.33 | 71 | 31 | 67.2 | 86 |
| 22 | Belgium | bel | EUR |  | 43585 | 0.812 | 7.26 | 75 | 65 | 64.2 | 59 |
| 23 | Malaysia | mys | APAC | CWLTH | 26315 | 0.671 | 7.22 | 26 | 100 | 9.4 | 62.57 |
| 24 | Greece | grc | EUR |  | 26449 | 0.797 | 6.87 | 35 | 60 | 88.1 | 51 |
| 25 | Portugal | prt | EUR |  | 27835 | 0.728 | 7.42 | 27 | 63 | 93.8 | 27 |
| 26 | Czech Republic | cze | EUR |  | 31549 | 0.866 | 7.33 | 58 | 57 | 23.3 | 27 |
| 27 | Mexico | mex | NAM |  | 17534 | 0.638 | 6.79 | 30 | 81 | 95.1 | 12.9 |
| 28 | Denmark | dnk | EUR |  | 45709 | 0.873 | 7.58 | 74 | 18 | 83.5 | 86 |
| 29 | Israel | isr | MEA |  | 33656 | 0.854 | 7.38 | 54 | 13 | 2 | 84.97 |
| 30 | Austria | aut | EUR |  | 47250 | 0.794 | 7.46 | 55 | 11 | 80.4 | 73 |
| 31 | Singapore | sgp | APAC | CWLTH | 85253 | 0.768 | 8.52 | 20 | 74 | 18.2 | 80 |
| 32 | Finland | fin | EUR |  | 41120 | 0.815 | 7.61 | 63 | 33 | 81.6 | 70 |
| 33 | Norway | nor | EUR |  | 68430 | 0.91 | 7.51 | 69 | 31 | 84.7 | 90 |
| 34 | South Africa | zaf | AFR | CWLTH | 13165 | 0.695 | 6.74 | 65 | 49 | 81.2 | 31 |
| 35 | Romania | rou | EUR |  | 20787 | 0.748 | 7.69 | 30 | 90 | 99.5 | 31 |
| 36 | Hong Kong | hkg | APAC |  | 56701 | 0.767 | 8.97 | 25 | 68 | 14.3 | 46.07 |
| 37 | New Zealand | nzl | APAC | CWLTH | 36172 | 0.917 | 8.19 | 79 | 22 | 57 | 97.82 |
| 38 | Egypt | egy | AFR |  | 11850 | 0.573 | 6.34 | 25 | 70 | 5.1 | 35 |
| 39 | Thailand | tha | APAC |  | 16097 | 0.608 | 6.63 | 20 | 64 | 0.9 | 27.16 |
| 40 | Argentina | arg | SAM |  | 22554 | 0.783 | 5.2 | 46 | 49 | 85.2 | 6.52 |
| 41 | Ireland | irl | EUR |  | 55533 | 0.887 | 7.9 | 70 | 28 | 92 | 98.37 |
| 42 | Pakistan | pak | APAC | CWLTH | 5000 | 0.302 | 6.28 | 14 | 55 | 1.6 | 49 |
| 43 | Hungary | hun | EUR |  | 26222 | 0.805 | 7.25 | 80 | 46 | 81 | 20 |
| 44 | Saudi Arabia | sau | MEA |  | 53624 | 0.723 | 6.95 | 25 | 95 | 4.4 |  |
| 45 | Ukraine | ukr | EUR | PSOV | 7519 | 0.796 | 6.2 | 25 | 92 | 83.8 |  |
| 46 | Chile | chl | SAM |  | 23460 | 0.746 | 7.87 | 23 | 63 | 89.4 | 9.53 |
| 47 | Serbia | srb | EUR |  | 13671 | 0.695 | 6.65 | 25 | 86 | 92.5 |  |
| 48 | Croatia | hrv | EUR |  | 21581 | 0.77 | 6.91 | 33 | 73 | 93.4 | 49 |
| 49 | Nigeria | nga | AFR | CWLTH | 6108 | 0.198 | 6.44 | 30 | 80 | 49.3 | 53 |
| 50 | Tunisia | tun | AFR |  | 11428 | 0.621 | 6.39 |  |  | 0.2 |  |
| 51 | Colombia | col | SAM |  | 14847 | 0.602 | 6.56 | 13 | 67 | 92.5 | 4.22 |
| 52 | Slovenia | svn | EUR |  | 31007 | 0.863 | 6.44 | 27 | 71 | 78.4 | 59 |
| 53 | Slovakia | svk | EUR |  | 29720 | 0.802 | 7.29 | 52 | 100 | 85.3 | 26 |
| 54 | Algeria | dza | AFR |  | 14504 | 0.643 | 5.2 |  |  | 0.2 | 7 |
| 55 | Bulgaria | bgr | EUR |  | 19097 | 0.749 | 7.33 | 30 | 70 | 82.1 | 25 |
| 56 | Morocco | mar | AFR |  | 8164 | 0.468 | 6.46 | 46 | 70 | 0.06 | 14 |
| 57 | Lithuania | ltu | EUR | PSOV | 28359 | 0.877 | 7.61 | 60 | 42 | 89.8 | 38 |
| 58 | Indonesia | idn | APAC |  | 11126 | 0.603 | 7.01 | 14 | 78 | 9.9 |  |
| 59 | Bangladesh | bgd | APAC | CWLTH | 3607 | 0.447 | 6.42 | 20 | 80 | 0.2 | 18 |
| 60 | Jordan | jor | MEA |  | 12123 | 0.7 | 7.93 | 30 | 70 | 2.2 | 45 |
| 61 | United Arab Emirates | are | MEA |  | 67617 | 0.673 |  | 25 | 90 | 12.6 |  |
| 62 | Estonia | est | EUR | PSOV | 28592 | 0.859 | 7.58 | 60 | 40 | 39.9 | 50 |
| 63 | Viet Nam | vnm | APAC |  | 6024 | 0.513 | 6.46 | 20 | 70 | 4.8 |  |
| 64 | Cuba | cub | NAM |  | 20611 | 0.743 |  |  |  | 59.2 |  |
| 65 | Venezuela | ven | SAM |  | 16673 | 0.682 | 3.23 | 12 | 81 | 89.3 |  |
| 66 | Latvia | lva | EUR | PSOV | 24712 | 0.813 | 7.42 | 70 | 44 | 55.8 | 46 |
| 67 | Lebanon | lbn | MEA |  | 18240 | 0.631 | 7.01 | 40 | 75 | 38.3 | 40 |
| 68 | Belarus | blr | EUR | PSOV | 17654 | 0.82 |  |  |  | 71.2 |  |
| 69 | Cyprus | cyp | EUR | CWLTH | 32785 | 0.776 | 7.03 |  |  | 73.2 | 73 |
| 70 | Kenya | ken | AFR | CWLTH | 3208 | 0.515 | 7.16 | 25 | 70 | 84.8 | 18.83 |
| 71 | Philippines | phl | APAC |  | 7254 | 0.61 | 7.14 | 32 | 94 | 92.6 | 92 |
| 72 | Iraq | irq | MEA |  | 15474 | 0.467 |  | 30 | 95 | 0.8 | 35 |
| 73 | Kuwait | kwt | MEA |  | 70166 | 0.646 | 7.46 | 25 | 90 | 14.3 |  |
| 74 | Ethiopia | eth | AFR |  | 1801 | 0.317 | 5.68 | 20 | 70 | 62.8 |  |
| 75 | Oman | omn | MEA |  | 44628 | 0.603 | 7.21 |  |  | 6.5 |  |
| 76 | Iceland | isl | EUR |  | 46097 | 0.847 | 6.87 | 60 | 30 | 95 |  |
| 77 | Luxembourg | lux | EUR |  | 98987 | 0.762 | 7.51 | 60 | 40 | 70.4 | 56 |
| 78 | Peru | per | SAM |  | 12195 | 0.664 | 7.34 | 16 | 64 | 93 |  |
| 79 | Sri Lanka | lka | APAC | CWLTH | 10566 | 0.738 | 6.57 | 35 | 80 | 7.3 | 9.9 |
| 80 | Uruguay | ury | SAM |  | 21507 | 0.712 | 7.18 | 36 | 61 | 57.9 |  |
| 81 | Qatar | qat | MEA |  | 132099 | 0.686 | 7.77 |  |  | 13.8 |  |
| 82 | Ghana | gha | AFR | CWLTH | 4266 | 0.553 | 6.2 | 15 | 80 | 74.9 | 66.67 |
| 83 | Armenia | arm | MEA | PSOV | 8468 | 0.701 | 7.67 |  |  | 98.5 |  |
| 84 | Kazakhstan | kaz | APAC | PSOV | 24268 | 0.762 | 7.26 |  |  | 24.8 | 15.4 |
| 85 | Azerbaijan | aze | MEA | PSOV | 17993 | 0.7 | 6.34 |  |  | 3 |  |
| 86 | Uganda | uga | AFR | CWLTH | 2003 | 0.479 | 7.3 |  |  | 86.7 | 8.09 |
| 87 | Cameroon | cmr | AFR | CWLTH | 3144 | 0.486 | 6.34 |  |  | 70.3 | 38 |
| 88 | Puerto Rico | pri | NAM |  | 34938 |  |  |  |  | 96.7 | 48.61 |
| 89 | Bosnia & Herzegovina | bih | EUR |  | 10492 | 0.655 | 6.98 |  |  | 52.3 |  |
| 90 | Georgia | geo | APAC | PSOV | 9630 | 0.77 | 7.83 |  |  | 88.5 |  |
| 91 | Tanzania | tza | AFR | CWLTH | 2904 | 0.426 | 6.92 | 25 | 70 | 61.4 | 9.89 |
| 92 | Nepal | npl | APAC |  | 2465 | 0.452 | 6.56 | 30 | 65 | 0.5 | 46.49 |
| 93 | Macedonia | mkd | EUR |  | 14009 |  | 7.19 |  |  | 59.3 |  |
| 94 | Sudan | sdn | AFR |  | 4344 | 0.306 |  |  |  | 5.4 |  |
| 95 | Macao | mac | APAC |  | 139767 |  |  |  |  | 7.2 |  |
| 96 | Uzbekistan | uzb | APAC | PSOV | 6068 | 0.651 |  |  |  | 2.3 |  |
| 97 | Syria | syr | MEA |  | 5100 | 0.553 | 5.19 | 35 | 80 | 5.2 |  |
| 98 | Costa Rica | cri | NAM |  | 15482 | 0.654 | 7.53 | 15 | 35 | 90.9 |  |
| 99 | Senegal | sen | AFR |  | 2451 | 0.368 | 6.32 | 25 | 70 | 3.7 |  |
| 100 | Malta | mlt | EUR | CWLTH | 35826 | 0.733 | 7.61 | 59 | 56 | 97 | 89 |
| 101 | Palestine | pse | MEA |  | 4900 | 0.662 |  |  |  | 2.4 |  |
| 102 | Ecuador | ecu | SAM |  | 11264 | 0.594 | 5.99 | 8 | 78 | 94.1 |  |
| 103 | Libya | lby | AFR |  | 14650 | 0.698 |  | 38 | 80 | 2.7 |  |
| 104 | Botswana | bwa | AFR | CWLTH | 16368 | 0.619 | 7.13 |  |  | 72.1 | 38.42 |
| 105 | Trinidad & Tobago | tto | NAM | CWLTH | 32635 | 0.7 | 7.07 | 16 | 47 | 65.9 | 87.74 |
| 106 | Zimbabwe | zwe | AFR |  | 2096 | 0.5 | 5.33 |  |  | 87 | 41.58 |
| 107 | Bahrain | bhr | MEA |  | 50095 | 0.714 | 7.55 |  |  | 14.5 |  |
| 108 | Jamaica | jam | NAM | CWLTH | 8759 | 0.668 | 7.33 | 39 | 45 | 77.2 | 97.64 |
| 109 | Cote d'Ivoire | civ | AFR |  | 3316 | 0.389 | 6.03 |  |  | 44.1 |  |
| 110 | Albania | alb | EUR |  | 11301 | 0.609 | 7.18 | 20 | 90 | 18 |  |

a – AFR: Africa; APAC: Asia-Pacific; EUR: Europe; MEA: Middle East; NAM: North America; SAM: South America; CWLTH: Commonwealth; PSOV: Post-Soviet States
b – estimated using value from Hong Kong
c – estimated as 1%, roughly the value for China
