# Supplementary material for: The Research Focus of Nations: Economic vs. Altruistic Motivations
Source: PLoS One. 2017 Jan 5;12(1):e0169383. doi: 10.1371/journal.pone.0169383 (PMC5215941; doi:10.1371/journal.pone.0169383)
Supplement: S2 Table — (DOCX) [file pone.0169383.s002.docx]

**Table S2. Description of 114 DC2 disciplines and grouping into fields.**

| **Field** | **DC2** | **Label** | **Coeff** | **#Doc** | **%Ind** |
| --- | --- | --- | --- | --- | --- |
| 1 - Civics | 22 | Medieval Studies | 0.664 | 73615 | 0.77% |
| 1 - Civics | 0 | Governance | 0.844 | 255756 | 0.97% |
| 1 - Civics | 95 | Philosophy | 0.631 | 22448 | 1.26% |
| 1 - Civics | 14 | Learning | 0.730 | 143874 | 1.79% |
| 1 - Civics | 65 | Archaeology | 0.345 | 30618 | 2.62% |
| 1 - Civics | 2 | Psychiatry | 0.624 | 216210 | 2.95% |
| 1 - Civics | 103 | Information Science | 0.445 | 23262 | 3.05% |
| 1 - Civics | 99 | Law | 0.477 | 18757 | 3.41% |
| 1 - Civics | 13 | Economics & Finance | 0.548 | 152004 | 3.51% |
| 1 - Civics | 39 | Brain, Vision & Hearing | 0.223 | 94128 | 3.97% |
| 1 - Civics | 12 | Management | 0.684 | 182983 | 4.39% |
| 1 - Civics | 17 | Patient Care | 0.354 | 152943 | 4.40% |
| 1 - Civics | 90 | Sound | 0.228 | 31554 | 8.19% |
| 2 - Medicine | 62 | Pregnancy & Childbirth | 0.397 | 52615 | 2.73% |
| 2 - Medicine | 71 | Emergency Medicine | 0.701 | 50190 | 2.95% |
| 2 - Medicine | 75 | Neurology | 0.599 | 45265 | 2.99% |
| 2 - Medicine | 37 | Gastrointestinal Science | 0.727 | 90302 | 3.06% |
| 2 - Medicine | 91 | Reconstructive Surgery | 0.588 | 29158 | 3.58% |
| 2 - Medicine | 44 | Dentistry | 0.182 | 81699 | 3.61% |
| 2 - Medicine | 74 | Endocrinology | 0.694 | 39253 | 3.85% |
| 2 - Medicine | 24 | Orthopedics | 0.424 | 131779 | 3.87% |
| 2 - Medicine | 70 | Reproductive Medicine | 0.203 | 39309 | 4.10% |
| 2 - Medicine | 35 | Oncology | 0.732 | 109957 | 4.14% |
| 2 - Medicine | 49 | Cancer in Women | 0.541 | 66723 | 4.21% |
| 2 - Medicine | 57 | Ophthalmology | 0.234 | 58620 | 4.22% |
| 2 - Medicine | 60 | Urology | 0.658 | 58190 | 4.26% |
| 2 - Medicine | 80 | Sleep | 0.466 | 41101 | 4.27% |
| 2 - Medicine | 26 | Cardiology | 0.537 | 130550 | 4.33% |
| 2 - Medicine | 46 | Liver Diseases | 0.564 | 70138 | 4.53% |
| 2 - Medicine | 87 | Bone Science | 0.542 | 36589 | 5.42% |
| 2 - Medicine | 76 | Anesthesiology | 0.652 | 39056 | 5.53% |
| 2 - Medicine | 101 | Blood Disorders | 0.394 | 19026 | 5.74% |
| 2 - Medicine | 8 | Neurodegenerative Diseases | 0.355 | 163842 | 5.87% |
| 2 - Medicine | 18 | Diabetes | 0.538 | 154828 | 6.02% |
| 2 - Medicine | 11 | Immunology | 0.674 | 163067 | 6.12% |
| 2 - Medicine | 50 | Hematology | 0.547 | 57963 | 6.32% |
| 2 - Medicine | 69 | Dermatology | 0.540 | 48454 | 6.60% |
| 2 - Medicine | 111 | Tobacco | 0.146 | 12765 | 8.42% |
| 2 - Medicine | 55 | Respiratory Diseases | 0.451 | 49942 | 8.67% |
| 2 - Medicine | 106 | Medical Imaging | 0.186 | 16580 | 9.22% |
| 3 - Inf Disease | 98 | Tuberculosis | 0.668 | 25011 | 3.65% |
| 3 - Inf Disease | 42 | Tropical Disease & Parasites | 0.727 | 65643 | 4.63% |
| 3 - Inf Disease | 107 | Mycology | 0.228 | 15417 | 4.70% |
| 3 - Inf Disease | 63 | AIDS | 0.594 | 58606 | 6.68% |
| 3 - Inf Disease | 40 | Microbiology | 0.727 | 83820 | 6.74% |
| 3 - Inf Disease | 92 | Veterinary Sciences | 0.210 | 21046 | 7.03% |
| 3 - Inf Disease | 78 | Virology | 0.773 | 46339 | 7.49% |
| 4 - Sustainability | 104 | Brazil | -0.094 | 19690 | 0.81% |
| 4 - Sustainability | 21 | Ecology | -0.584 | 106398 | 4.14% |
| 4 - Sustainability | 31 | Entomology | -0.504 | 72687 | 4.20% |
| 4 - Sustainability | 30 | Wildlife Science | -0.436 | 77716 | 4.93% |
| 4 - Sustainability | 5 | Marine Science | -0.442 | 125972 | 5.07% |
| 4 - Sustainability | 3 | Plant Science | -0.474 | 155207 | 5.37% |
| 4 - Sustainability | 61 | Agricultural Policy | -0.323 | 61387 | 6.42% |
| 4 - Sustainability | 29 | Animal Science | -0.328 | 91264 | 8.19% |
| 4 - Sustainability | 54 | Climate Science | -0.227 | 56089 | 8.29% |
| 4 - Sustainability | 81 | Environmental Engineering | -0.383 | 34840 | 8.42% |
| 4 - Sustainability | 4 | Geoscience | -0.307 | 116291 | 11.47% |
| 4 - Sustainability | 100 | Forestry | -0.114 | 17502 | 12.27% |
| 5 - Biochemistry | 1 | Molecular Chemistry | 0.397 | 196050 | 4.43% |
| 5 - Biochemistry | 110 | Russian Biochemistry | 0.103 | 9259 | 4.68% |
| 5 - Biochemistry | 97 | Alcohol | 0.337 | 18839 | 5.43% |
| 5 - Biochemistry | 36 | Medicinal Chemistry | 0.494 | 103266 | 5.48% |
| 5 - Biochemistry | 79 | Receptor & Channel Science | 0.341 | 31733 | 5.62% |
| 5 - Biochemistry | 7 | Cell Biology | 0.400 | 219759 | 5.95% |
| 5 - Biochemistry | 73 | Toxicology | 0.206 | 35910 | 6.47% |
| 5 - Biochemistry | 19 | Molecular Biochemistry | 0.547 | 133888 | 7.09% |
| 5 - Biochemistry | 82 | Metabolism Science | 0.484 | 32900 | 7.66% |
| 5 - Biochemistry | 51 | Environmental Chemistry | 0.219 | 53865 | 8.03% |
| 5 - Biochemistry | 77 | Analytical Chemistry | 0.444 | 42550 | 12.60% |
| 5 - Biochemistry | 68 | Pharmacology | 0.551 | 48998 | 14.27% |
| 6 - Basic Physics | 32 | Particle Physics | -0.200 | 85963 | 3.33% |
| 6 - Basic Physics | 48 | Astronomy & Astrophysics | -0.241 | 55490 | 6.09% |
| 6 - Basic Physics | 56 | Planetary Science | -0.288 | 44285 | 8.24% |
| 6 - Basic Physics | 58 | Nuclear Medicine | -0.186 | 53390 | 10.25% |
| 6 - Basic Physics | 94 | Plasma Physics | -0.170 | 28795 | 12.42% |
| 7 - Artif Intell | 20 | Mathematics | 0.161 | 124603 | 0.75% |
| 7 - Artif Intell | 102 | Nonlinear Dynamics | 0.150 | 24806 | 2.95% |
| 7 - Artif Intell | 45 | Operations Research | 0.459 | 98409 | 5.94% |
| 7 - Artif Intell | 72 | Statistics | 0.349 | 50757 | 6.14% |
| 7 - Artif Intell | 83 | Cryptography | 0.536 | 47570 | 6.95% |
| 7 - Artif Intell | 34 | Industrial Engineering | 0.439 | 134045 | 7.51% |
| 7 - Artif Intell | 9 | Computer Vision & Imaging | 0.689 | 248376 | 8.23% |
| 7 - Artif Intell | 67 | Human Computing | 0.481 | 67277 | 9.56% |
| 7 - Artif Intell | 27 | Networks | 0.635 | 166745 | 10.42% |
| 7 - Artif Intell | 6 | Computing | 0.678 | 212465 | 11.26% |
| 7 - Artif Intell | 52 | Telecommunications | 0.483 | 94400 | 12.48% |
| 8 - Appl Physics | 25 | Atomic Physics | 0.321 | 102140 | 6.13% |
| 8 - Appl Physics | 28 | Magnetics | 0.396 | 101358 | 7.81% |
| 8 - Appl Physics | 23 | Electrochemistry & Energy | 0.400 | 152670 | 7.98% |
| 8 - Appl Physics | 16 | Polymers | 0.349 | 125377 | 9.00% |
| 8 - Appl Physics | 41 | Nanochemistry | 0.437 | 109951 | 9.00% |
| 8 - Appl Physics | 66 | Optical Materials | 0.333 | 40798 | 9.14% |
| 8 - Appl Physics | 64 | Carbon Science | 0.520 | 66988 | 9.44% |
| 8 - Appl Physics | 38 | Optoelectronics & Photonics | 0.262 | 139783 | 12.25% |
| 8 - Appl Physics | 15 | Semiconductor Physics | 0.426 | 163864 | 14.15% |
| 8 - Appl Physics | 108 | Electronic Packaging | 0.232 | 15897 | 26.16% |
| 8 - Appl Physics | 113 | Lithography | 0.140 | 8684 | 33.77% |
| 9 - Engineering | 109 | Acoustic Engineering | 0.114 | 14053 | 8.28% |
| 9 - Engineering | 88 | Transportation | 0.375 | 37387 | 8.94% |
| 9 - Engineering | 59 | Composites | 0.390 | 58178 | 9.39% |
| 9 - Engineering | 33 | Fluid Mechanics | 0.342 | 108947 | 10.34% |
| 9 - Engineering | 85 | Separation Science | 0.451 | 41379 | 10.93% |
| 9 - Engineering | 89 | Mining Chemistry | 0.204 | 25412 | 11.20% |
| 9 - Engineering | 93 | Energy Usage | 0.433 | 38775 | 11.83% |
| 9 - Engineering | 47 | Civil Engineering | 0.522 | 89557 | 11.88% |
| 9 - Engineering | 96 | Machining & Tribology | 0.427 | 32842 | 13.50% |
| 9 - Engineering | 112 | Offshore Mechanics | 0.214 | 13331 | 15.03% |
| 9 - Engineering | 10 | Materials | 0.423 | 165706 | 15.72% |
| 9 - Engineering | 86 | Energy Production | 0.434 | 32972 | 16.38% |
| 9 - Engineering | 84 | Nuclear Science | 0.262 | 40533 | 16.59% |
| 9 - Engineering | 43 | Power & Electricity | 0.366 | 132275 | 17.91% |
| 9 - Engineering | 105 | Petroleum Engineering | 0.288 | 18289 | 18.61% |
| 9 - Engineering | 53 | Geological Engineering | 0.478 | 75681 | 20.24% |
