# Supplementary material for: The Research Focus of Nations: Economic vs. Altruistic Motivations
Source: PLoS One. 2017 Jan 5;12(1):e0169383. doi: 10.1371/journal.pone.0169383 (PMC5215941; doi:10.1371/journal.pone.0169383)
Supplement: S3 Table — Due to overlap in the matches, the unique set of matches is less than the sum over strings and match types. (DOCX) [file pone.0169383.s003.docx]

**Table S3. Industry-related strings used to find industry authored papers along with the numbers of matches for 2010-2013 using institution strings and associated Scopus affiliation profiles. Due to overlap in the matches, the unique set of matches is less than the sum over strings and match types.**

| **String** | **Representative Country** | **Similar Type** | **String Match** | **Affil Profile Match** |
| --- | --- | --- | --- | --- |
| % Ltd | US, UK |  | 278937 | 252526 |
| % Inc | US |  | 263238 | 311152 |
| %corporat% | US |  | 190577 | 246188 |
| %company% | US |  | 106073 | 105542 |
| % GmbH | Austria | Ltd | 86401 | 104413 |
| % Limited | UK |  | 36342 | 39154 |
| % LLC | US |  | 32093 | 24056 |
| % AG | Austria | PLC | 31815 | 52814 |
| %industries% | US, UK |  | 27966 | 23169 |
| % Corp | US |  | 23464 | 50128 |
| % SA | France | PLC | 19360 | 18499 |
| % Co | US |  | 19003 | 21923 |
| % AB | Sweden | Ltd | 10842 | 9827 |
| % Spa | Italy | PLC | 10610 | 10944 |
| % BV | Netherlands | Ltd | 9493 | 7968 |
| % A/S | Denmark | PLC | 8728 | 10253 |
| % SRL | Argentina | Ltd | 8362 | 5259 |
| % KG | Austria | LP | 7730 | 8083 |
| % AS | Estonia | PLC | 7430 | 8433 |
| % SAS | France | private corp | 5036 | 2441 |
| PT % | Indonesia | Ltd | 4015 | 4465 |
| % PLC | UK |  | 3970 | 4182 |
| %gGmbH | Austria | Ltd | 3407 | 3207 |
| % LLP | UK |  | 3399 | 1969 |
| % nv | Netherlands | PLC | 3133 | 4112 |
| % SE | EU | PLC | 3131 | 573 |
| %Co,Ltd | UK | Ltd | 2769 | 1546 |
| % SL | Spain | Ltd | 2526 | 1308 |
| % KK | Japan | Corp | 2397 | 2073 |
| % Ltda | Brazil | Ltd | 2250 | 1427 |
| %CoLtd | UK | Ltd | 2171 | 1517 |
| % oy | Sweden | Ltd | 2107 | 1637 |
| %GmbH) | Austria | Ltd | 1530 | 189 |
| % ASA | Norway | PLC | 1505 | 1560 |
| % sro | Czech | Ltd | 1380 | 565 |
| % KGaA | Germany | partnership | 1266 | 1617 |
| % BVBA | Belgium | Ltd | 1122 | 689 |
| % ApS | Denmark | PLC | 689 | 599 |
| % SARL | France | PLC | 638 | 314 |
| %Ltd) | UK | Ltd | 635 | 139 |
| % kft | Hungary | Ltd | 615 | 261 |
| % cv | Netherlands | LP | 609 | 334 |
| % S A | Guatemala | PLC | 580 | 459 |
| % GbR | Germany | partnership | 416 | 196 |
| % Lda | Portugal | Ltd | 371 | 117 |
| % OAO | Russia | PLC | 371 | 45 |
| % Oyj | Finland | PLC | 354 | 346 |
| % OHG | Germany | partnership | 297 | 338 |
| % Aktiengesellschaft | Austria | PLC | 230 | 1408 |
| % OG | Austria | GP | 220 | 134 |
| % OU | Estonia | LLC | 204 | 11 |
| % Coop | Phil | Coop | 183 | 118 |
| % S C | Guatemala | Soc Coll | 110 | 41 |
| % ehf | Iceland | Ltd | 81 | 45 |
| % stG | Austria | part by estoppel | 61 | 0 |
| % SF | Portugal | private corp | 59 | 26 |
| % SCS | Argentian | LP | 57 | 17 |
| % Kaisha | Japan | Corp | 57 | 29 |
| % AE | Greece | PLC | 46 | 38 |
| % I/S | Den | GP | 38 | 26 |
| % Tbk | Indonesia | PLC | 34 | 4 |
| % SEM | France | govt-owned corp | 30 | 4 |
| % GK | Japan | LLC | 27 | 12 |
| % vof | Netherlands | GP | 26 | 3 |
| % eK | Germany | SP | 26 | 12 |
| % BM | Israel | Ltd | 18 | 0 |
