# Supplementary material for: The Research Focus of Nations: Economic vs. Altruistic Motivations
Source: PLoS One. 2017 Jan 5;12(1):e0169383. doi: 10.1371/journal.pone.0169383 (PMC5215941; doi:10.1371/journal.pone.0169383)
Supplement: S4 Table — (DOCX) [file pone.0169383.s004.docx]

**Table S3. National publication fractions by field.**

| **Country** | **1**  **Civics** | **2**  **Med** | **3**  **Dis** | **4**  **Sust** | **5**  **Chem** | **6**  **BPhys** | **7**  **Comp** | **8**  **Aphys** | **9**  **Eng** | **Motiv** |
| --- | --- | --- | --- | --- | --- | --- | --- | --- | --- | --- |
| WORLD | 0.1567 | 0.2081 | 0.0354 | 0.1048 | 0.1039 | 0.0300 | 0.1423 | 0.1151 | 0.1037 | 4.65 |
| United States | 0.2262 | 0.2560 | 0.0414 | 0.0921 | 0.1009 | 0.0322 | 0.1026 | 0.0825 | 0.0662 | 3.90 |
| China | 0.0420 | 0.0949 | 0.0148 | 0.0946 | 0.1153 | 0.0218 | 0.2310 | 0.1727 | 0.2129 | 6.28 |
| United Kingdom | 0.3088 | 0.2350 | 0.0401 | 0.0860 | 0.0782 | 0.0284 | 0.0937 | 0.0637 | 0.0662 | 3.57 |
| Japan | 0.0546 | 0.2660 | 0.0234 | 0.0786 | 0.1247 | 0.0395 | 0.1303 | 0.1900 | 0.0931 | 5.10 |
| Germany | 0.1390 | 0.2301 | 0.0259 | 0.0889 | 0.1111 | 0.0506 | 0.1390 | 0.1366 | 0.0789 | 4.67 |
| India | 0.0562 | 0.1631 | 0.0486 | 0.1183 | 0.1865 | 0.0263 | 0.1506 | 0.1372 | 0.1132 | 5.26 |
| France | 0.1415 | 0.2135 | 0.0412 | 0.0952 | 0.0914 | 0.0410 | 0.1634 | 0.1223 | 0.0906 | 4.71 |
| Italy | 0.1204 | 0.2980 | 0.0313 | 0.1000 | 0.0958 | 0.0509 | 0.1377 | 0.0843 | 0.0817 | 4.37 |
| Canada | 0.2223 | 0.2168 | 0.0338 | 0.1220 | 0.0864 | 0.0235 | 0.1325 | 0.0741 | 0.0886 | 4.14 |
| Spain | 0.1696 | 0.2342 | 0.0422 | 0.1247 | 0.1038 | 0.0296 | 0.1386 | 0.0870 | 0.0703 | 4.26 |
| South Korea | 0.0577 | 0.2192 | 0.0269 | 0.0609 | 0.1247 | 0.0214 | 0.1718 | 0.2052 | 0.1121 | 5.43 |
| Australia | 0.2798 | 0.2110 | 0.0383 | 0.1469 | 0.0636 | 0.0189 | 0.0970 | 0.0642 | 0.0802 | 3.75 |
| Brazil | 0.0802 | 0.2239 | 0.0690 | 0.2914 | 0.0931 | 0.0219 | 0.0884 | 0.0642 | 0.0679 | 4.24 |
| Taiwan | 0.1279 | 0.1647 | 0.0230 | 0.0519 | 0.0872 | 0.0147 | 0.2281 | 0.2040 | 0.0985 | 5.37 |
| Netherlands | 0.2350 | 0.2956 | 0.0389 | 0.0945 | 0.0781 | 0.0241 | 0.1026 | 0.0688 | 0.0625 | 3.69 |
| Iran | 0.0831 | 0.1664 | 0.0365 | 0.1092 | 0.1247 | 0.0225 | 0.1576 | 0.1191 | 0.1810 | 5.41 |
| Russian Fed | 0.0396 | 0.0678 | 0.0118 | 0.1094 | 0.1370 | 0.1040 | 0.1290 | 0.2633 | 0.1382 | 6.21 |
| Turkey | 0.1290 | 0.3535 | 0.0426 | 0.1004 | 0.0855 | 0.0167 | 0.1104 | 0.0684 | 0.0936 | 4.06 |
| Poland | 0.0763 | 0.2264 | 0.0300 | 0.1263 | 0.1122 | 0.0346 | 0.1380 | 0.1355 | 0.1206 | 5.03 |
| Switzerland | 0.1529 | 0.2523 | 0.0494 | 0.1064 | 0.1044 | 0.0556 | 0.1066 | 0.1056 | 0.0670 | 4.28 |
| Sweden | 0.1952 | 0.2497 | 0.0316 | 0.1083 | 0.0877 | 0.0285 | 0.1247 | 0.0848 | 0.0894 | 4.19 |
| Belgium | 0.2001 | 0.2356 | 0.0508 | 0.1009 | 0.0859 | 0.0337 | 0.1237 | 0.0977 | 0.0717 | 4.15 |
| Malaysia | 0.1760 | 0.0872 | 0.0250 | 0.1063 | 0.0891 | 0.0092 | 0.1975 | 0.1445 | 0.1652 | 5.38 |
| Greece | 0.1432 | 0.2812 | 0.0336 | 0.0976 | 0.0727 | 0.0265 | 0.1769 | 0.0723 | 0.0961 | 4.40 |
| Portugal | 0.1476 | 0.1623 | 0.0337 | 0.1319 | 0.1114 | 0.0293 | 0.1826 | 0.0987 | 0.1024 | 4.82 |
| Czech Republic | 0.1161 | 0.1929 | 0.0290 | 0.1381 | 0.1019 | 0.0346 | 0.1534 | 0.1161 | 0.1179 | 4.92 |
| Mexico | 0.1045 | 0.1685 | 0.0479 | 0.2073 | 0.0937 | 0.0411 | 0.1268 | 0.1231 | 0.0871 | 4.79 |
| Denmark | 0.1817 | 0.2865 | 0.0411 | 0.1180 | 0.1016 | 0.0219 | 0.0911 | 0.0877 | 0.0704 | 3.96 |
| Israel | 0.2280 | 0.2268 | 0.0322 | 0.0747 | 0.1025 | 0.0285 | 0.1579 | 0.1017 | 0.0477 | 4.11 |
| Austria | 0.1430 | 0.2381 | 0.0286 | 0.1035 | 0.0956 | 0.0315 | 0.1726 | 0.1010 | 0.0860 | 4.58 |
| Singapore | 0.1349 | 0.1424 | 0.0261 | 0.0377 | 0.1027 | 0.0055 | 0.2311 | 0.2260 | 0.0937 | 5.46 |
| Finland | 0.2208 | 0.1816 | 0.0241 | 0.1390 | 0.0745 | 0.0289 | 0.1747 | 0.0855 | 0.0709 | 4.30 |
| Norway | 0.2465 | 0.2034 | 0.0328 | 0.1634 | 0.0634 | 0.0166 | 0.1054 | 0.0478 | 0.1207 | 4.03 |
| South Africa | 0.2911 | 0.1084 | 0.0969 | 0.1808 | 0.0856 | 0.0242 | 0.0797 | 0.0513 | 0.0819 | 3.80 |
| Romania | 0.1544 | 0.1303 | 0.0129 | 0.0787 | 0.0837 | 0.0298 | 0.2156 | 0.1413 | 0.1534 | 5.39 |
| Hong Kong | 0.2280 | 0.1603 | 0.0264 | 0.0368 | 0.0853 | 0.0092 | 0.2245 | 0.1293 | 0.1002 | 4.76 |
| New Zealand | 0.2854 | 0.1987 | 0.0360 | 0.2156 | 0.0670 | 0.0096 | 0.0961 | 0.0378 | 0.0537 | 3.50 |
| Egypt | 0.0547 | 0.2354 | 0.0438 | 0.1182 | 0.1588 | 0.0229 | 0.1324 | 0.1049 | 0.1289 | 4.99 |
| Thailand | 0.0887 | 0.1675 | 0.0829 | 0.1419 | 0.1217 | 0.0098 | 0.1545 | 0.1267 | 0.1064 | 4.96 |
| Argentina | 0.1061 | 0.1780 | 0.0739 | 0.2916 | 0.1061 | 0.0328 | 0.0655 | 0.0925 | 0.0535 | 4.26 |
| Ireland | 0.2349 | 0.2281 | 0.0435 | 0.0945 | 0.0931 | 0.0164 | 0.1298 | 0.1028 | 0.0570 | 4.01 |
| Pakistan | 0.0995 | 0.2066 | 0.0579 | 0.1852 | 0.1298 | 0.0286 | 0.1468 | 0.0704 | 0.0754 | 4.52 |
| Hungary | 0.1441 | 0.2025 | 0.0299 | 0.1388 | 0.1204 | 0.0374 | 0.1577 | 0.0927 | 0.0766 | 4.56 |
| Saudi Arabia | 0.0797 | 0.1980 | 0.0447 | 0.0927 | 0.1257 | 0.0139 | 0.1915 | 0.1263 | 0.1274 | 5.19 |
| Ukraine | 0.0367 | 0.0383 | 0.0053 | 0.0572 | 0.1061 | 0.0889 | 0.2021 | 0.3036 | 0.1619 | 6.72 |
| Chile | 0.2084 | 0.1880 | 0.0426 | 0.2000 | 0.0876 | 0.0602 | 0.0956 | 0.0540 | 0.0636 | 3.98 |
| Serbia | 0.1130 | 0.2127 | 0.0251 | 0.1260 | 0.1073 | 0.0262 | 0.1816 | 0.1036 | 0.1045 | 4.85 |
| Croatia | 0.2340 | 0.2534 | 0.0360 | 0.1216 | 0.0872 | 0.0247 | 0.1099 | 0.0397 | 0.0934 | 3.85 |
| Nigeria | 0.1959 | 0.1768 | 0.1066 | 0.2175 | 0.1315 | 0.0130 | 0.0449 | 0.0255 | 0.0884 | 3.79 |
| Tunisia | 0.0699 | 0.1952 | 0.0480 | 0.1267 | 0.0995 | 0.0053 | 0.2668 | 0.0947 | 0.0940 | 5.11 |
| Colombia | 0.1871 | 0.1688 | 0.0695 | 0.1814 | 0.0716 | 0.0143 | 0.1149 | 0.0885 | 0.1038 | 4.35 |
| Slovenia | 0.2122 | 0.1452 | 0.0295 | 0.1179 | 0.1005 | 0.0302 | 0.1367 | 0.1030 | 0.1246 | 4.65 |
| Slovakia | 0.1307 | 0.1572 | 0.0294 | 0.1350 | 0.0986 | 0.0315 | 0.1741 | 0.1059 | 0.1377 | 5.06 |
| Algeria | 0.0344 | 0.0346 | 0.0129 | 0.0877 | 0.0689 | 0.0295 | 0.2830 | 0.1773 | 0.2716 | 6.86 |
| Bulgaria | 0.0775 | 0.1680 | 0.0336 | 0.1439 | 0.1302 | 0.0473 | 0.1420 | 0.1785 | 0.0789 | 5.16 |
| Morocco | 0.0602 | 0.2741 | 0.0604 | 0.0782 | 0.0863 | 0.0132 | 0.2289 | 0.0941 | 0.1047 | 4.91 |
| Lithuania | 0.1436 | 0.0927 | 0.0195 | 0.1266 | 0.0670 | 0.0256 | 0.1253 | 0.1659 | 0.2338 | 5.69 |
| Indonesia | 0.1649 | 0.0641 | 0.0381 | 0.2018 | 0.0723 | 0.0192 | 0.1991 | 0.0864 | 0.1543 | 5.16 |
| Bangladesh | 0.0980 | 0.0999 | 0.0731 | 0.1659 | 0.1456 | 0.0276 | 0.1732 | 0.1181 | 0.0985 | 5.12 |
| Jordan | 0.2025 | 0.1565 | 0.0282 | 0.0895 | 0.0877 | 0.0125 | 0.2436 | 0.0666 | 0.1131 | 4.73 |
| United Arab Em. | 0.2261 | 0.1250 | 0.0278 | 0.0700 | 0.0465 | 0.0074 | 0.2079 | 0.0700 | 0.2192 | 5.11 |
| Estonia | 0.2239 | 0.0861 | 0.0162 | 0.2025 | 0.0862 | 0.0242 | 0.1486 | 0.0999 | 0.1125 | 4.68 |
| Viet Nam | 0.0729 | 0.0458 | 0.0636 | 0.1266 | 0.0784 | 0.0224 | 0.3686 | 0.1365 | 0.0853 | 5.83 |
| Cuba | 0.1245 | 0.3466 | 0.1015 | 0.1797 | 0.1062 | 0.0118 | 0.0617 | 0.0311 | 0.0368 | 3.46 |
| Venezuela | 0.1140 | 0.1937 | 0.0927 | 0.2370 | 0.0920 | 0.0223 | 0.0865 | 0.0801 | 0.0816 | 4.30 |
| Latvia | 0.1085 | 0.0888 | 0.0154 | 0.1630 | 0.0905 | 0.0200 | 0.1787 | 0.1736 | 0.1615 | 5.65 |
| Lebanon | 0.1823 | 0.3059 | 0.0388 | 0.0514 | 0.0555 | 0.0139 | 0.2056 | 0.0435 | 0.1031 | 4.19 |
| Belarus | 0.0216 | 0.0531 | 0.0097 | 0.0476 | 0.0933 | 0.0736 | 0.1693 | 0.4012 | 0.1306 | 6.83 |
| Cyprus | 0.3432 | 0.1026 | 0.0115 | 0.0650 | 0.0597 | 0.0271 | 0.2148 | 0.0651 | 0.1109 | 4.33 |
| Kenya | 0.2289 | 0.1053 | 0.2400 | 0.3155 | 0.0475 | 0.0073 | 0.0241 | 0.0127 | 0.0187 | 3.14 |
| Philippines | 0.2891 | 0.1276 | 0.0636 | 0.2737 | 0.0638 | 0.0062 | 0.0872 | 0.0409 | 0.0477 | 3.55 |
| Iraq | 0.0515 | 0.1608 | 0.0540 | 0.1105 | 0.1125 | 0.0207 | 0.1731 | 0.1564 | 0.1605 | 5.57 |
| Kuwait | 0.1518 | 0.2272 | 0.0626 | 0.0937 | 0.0728 | 0.0103 | 0.1609 | 0.0363 | 0.1844 | 4.67 |
| Ethiopia | 0.1416 | 0.1187 | 0.2792 | 0.3138 | 0.0701 | 0.0055 | 0.0308 | 0.0225 | 0.0178 | 3.41 |
| Oman | 0.1577 | 0.2174 | 0.0353 | 0.1453 | 0.0777 | 0.0108 | 0.1502 | 0.0574 | 0.1483 | 4.58 |
| Iceland | 0.2138 | 0.2094 | 0.0424 | 0.2033 | 0.0711 | 0.0175 | 0.1387 | 0.0569 | 0.0468 | 3.88 |
| Luxembourg | 0.2032 | 0.1049 | 0.0225 | 0.0797 | 0.0790 | 0.0131 | 0.3485 | 0.0783 | 0.0709 | 4.98 |
| Peru | 0.2277 | 0.1641 | 0.2313 | 0.2155 | 0.0437 | 0.0143 | 0.0484 | 0.0217 | 0.0334 | 3.23 |
| Sri Lanka | 0.1855 | 0.1892 | 0.0818 | 0.1925 | 0.0905 | 0.0055 | 0.1542 | 0.0399 | 0.0607 | 4.01 |
| Uruguay | 0.1198 | 0.1871 | 0.0780 | 0.2590 | 0.1272 | 0.0142 | 0.1296 | 0.0535 | 0.0316 | 4.10 |
| Qatar | 0.1461 | 0.2428 | 0.0349 | 0.0395 | 0.0528 | 0.0056 | 0.2520 | 0.0745 | 0.1519 | 4.92 |
| Ghana | 0.2890 | 0.1171 | 0.1171 | 0.2397 | 0.0758 | 0.0273 | 0.0297 | 0.0191 | 0.0853 | 3.50 |
| Armenia | 0.0570 | 0.0814 | 0.0101 | 0.0387 | 0.1325 | 0.1645 | 0.1589 | 0.2779 | 0.0790 | 6.10 |
| Kazakhstan | 0.2484 | 0.0310 | 0.0290 | 0.0787 | 0.0754 | 0.1038 | 0.1456 | 0.1413 | 0.1468 | 5.18 |
| Azerbaijan | 0.0500 | 0.1389 | 0.0292 | 0.0648 | 0.1221 | 0.0261 | 0.2163 | 0.2398 | 0.1127 | 5.89 |
| Uganda | 0.2390 | 0.1385 | 0.3502 | 0.1925 | 0.0308 | 0.0033 | 0.0256 | 0.0028 | 0.0174 | 2.87 |
| Cameroon | 0.1116 | 0.1296 | 0.1668 | 0.2263 | 0.1531 | 0.0137 | 0.1017 | 0.0455 | 0.0517 | 4.17 |
| Puerto Rico | 0.1106 | 0.2147 | 0.0637 | 0.1714 | 0.1198 | 0.0194 | 0.0689 | 0.1805 | 0.0511 | 4.52 |
| Bosnia & Herzegovina | 0.2013 | 0.2719 | 0.0373 | 0.0845 | 0.0491 | 0.0190 | 0.2037 | 0.0258 | 0.1074 | 4.15 |
| Georgia | 0.1274 | 0.0887 | 0.0265 | 0.1095 | 0.1004 | 0.0937 | 0.2673 | 0.1133 | 0.0731 | 5.32 |
| Tanzania | 0.2088 | 0.1660 | 0.2977 | 0.2283 | 0.0369 | 0.0031 | 0.0151 | 0.0084 | 0.0357 | 3.04 |
| Nepal | 0.1564 | 0.3908 | 0.1350 | 0.1831 | 0.0541 | 0.0066 | 0.0135 | 0.0281 | 0.0325 | 3.00 |
| Macedonia | 0.1618 | 0.1610 | 0.0334 | 0.0886 | 0.0844 | 0.0150 | 0.2967 | 0.0695 | 0.0897 | 4.89 |
| Sudan | 0.0888 | 0.1452 | 0.2026 | 0.2559 | 0.0943 | 0.0251 | 0.1111 | 0.0295 | 0.0476 | 4.07 |
| Macao | 0.2736 | 0.0537 | 0.0147 | 0.0223 | 0.0894 | 0.0095 | 0.3854 | 0.0563 | 0.0950 | 5.02 |
| Uzbekistan | 0.0288 | 0.0494 | 0.0120 | 0.1200 | 0.1492 | 0.0923 | 0.1879 | 0.2469 | 0.1135 | 6.26 |
| Syria | 0.0848 | 0.1692 | 0.0387 | 0.2796 | 0.1301 | 0.0484 | 0.0566 | 0.0943 | 0.0985 | 4.63 |
| Costa Rica | 0.1559 | 0.0928 | 0.0742 | 0.4568 | 0.0924 | 0.0193 | 0.0581 | 0.0196 | 0.0310 | 3.81 |
| Senegal | 0.1216 | 0.2261 | 0.2234 | 0.1695 | 0.0826 | 0.0023 | 0.0986 | 0.0413 | 0.0348 | 3.68 |
| Malta | 0.2916 | 0.2575 | 0.0276 | 0.0840 | 0.0246 | 0.0204 | 0.1945 | 0.0190 | 0.0809 | 3.71 |
| Palestine | 0.1857 | 0.1169 | 0.0473 | 0.1105 | 0.1312 | 0.0186 | 0.1605 | 0.1513 | 0.0781 | 4.81 |
| Ecuador | 0.1470 | 0.1592 | 0.1000 | 0.4004 | 0.0427 | 0.0079 | 0.0760 | 0.0388 | 0.0281 | 3.72 |
| Libya | 0.0693 | 0.1766 | 0.0522 | 0.1039 | 0.1409 | 0.0171 | 0.1662 | 0.0848 | 0.1890 | 5.34 |
| Botswana | 0.4296 | 0.0647 | 0.1296 | 0.1572 | 0.0594 | 0.0045 | 0.0858 | 0.0208 | 0.0485 | 3.10 |
| Trinidad & Tobago | 0.2595 | 0.2032 | 0.0950 | 0.2118 | 0.0485 | 0.0086 | 0.0532 | 0.0297 | 0.0905 | 3.52 |
| Zimbabwe | 0.3364 | 0.0636 | 0.2139 | 0.3165 | 0.0288 | 0.0008 | 0.0148 | 0.0083 | 0.0170 | 2.84 |
| Bahrain | 0.2436 | 0.2645 | 0.0375 | 0.0446 | 0.0371 | 0.0105 | 0.1620 | 0.1136 | 0.0865 | 4.13 |
| Jamaica | 0.3308 | 0.2990 | 0.0693 | 0.1035 | 0.0900 | 0.0065 | 0.0638 | 0.0117 | 0.0254 | 2.81 |
| Cote d'Ivoire | 0.1035 | 0.2118 | 0.1986 | 0.2592 | 0.1132 | 0.0104 | 0.0451 | 0.0230 | 0.0352 | 3.60 |
| Albania | 0.2860 | 0.1494 | 0.0543 | 0.1949 | 0.0494 | 0.0264 | 0.1544 | 0.0158 | 0.0694 | 3.76 |
